# Global forest management data for 2015 at a 100 m resolution

**DOI:** 10.1038/s41597-022-01332-3

**Published:** 2022-05-10

**Authors:** Myroslava Lesiv, Dmitry Schepaschenko, Marcel Buchhorn, Linda See, Martina Dürauer, Ivelina Georgieva, Martin Jung, Florian Hofhansl, Katharina Schulze, Andrii Bilous, Volodymyr Blyshchyk, Liudmila Mukhortova, Carlos Luis Muñoz Brenes, Leonid Krivobokov, Stephan Ntie, Khongor Tsogt, Stephan Alexander Pietsch, Elena Tikhonova, Moonil Kim, Fulvio Di Fulvio, Yuan-Fong Su, Roma Zadorozhniuk, Flavius Sorin Sirbu, Kripal Panging, Svitlana Bilous, Sergii B. Kovalevskii, Florian Kraxner, Ahmed Harb Rabia, Roman Vasylyshyn, Rekib Ahmed, Petro Diachuk, Serhii S. Kovalevskyi, Khangsembou Bungnamei, Kusumbor Bordoloi, Andrii Churilov, Olesia Vasylyshyn, Dhrubajyoti Sahariah, Anatolii P. Tertyshnyi, Anup Saikia, Žiga Malek, Kuleswar Singha, Roman Feshchenko, Reinhard Prestele, Ibrar ul Hassan Akhtar, Kiran Sharma, Galyna Domashovets, Seth A. Spawn-Lee, Oleksii Blyshchyk, Oleksandr Slyva, Mariia Ilkiv, Oleksandr Melnyk, Vitalii Sliusarchuk, Anatolii Karpuk, Andrii Terentiev, Valentin Bilous, Kateryna Blyshchyk, Maxim Bilous, Nataliia Bogovyk, Ivan Blyshchyk, Sergey Bartalev, Mikhail Yatskov, Bruno Smets, Piero Visconti, Ian Mccallum, Michael Obersteiner, Steffen Fritz

**Affiliations:** 1 International Institute for Applied Systems Analysis, Laxenburg, A-2361 Austria; 2 V.N. Sukachev Institute of Forest, Siberian Branch of the Russian Academy of Science, Akademgorodok 50(28), Krasnoyarsk, 660036 Russia; 3 Remote Sensing Unit, Flemish Institute for Technological Research (VITO), B-2400 Mol, Belgium; 4 Department of Environmental Geography, Institute for Environmental Studies (IVM), Vrije Universiteit Amsterdam, De Boelelaan 1111, 1081 HV Amsterdam, the Netherlands; 5 National University of Life and Environmental Sciences of Ukraine, Heroiv Oborony street 15, Kyiv, 03041 Ukraine; 6 Moore Center for Science, Conservation International, 2011 Crystal Drive, Suite 600, Arlington, VA 22202 USA; 7 Université des Sciences et Techniques de Masuku, Laboratoire de Biologie Moléculaire et Cellulaire (LABMC), Département de Biologie, Université des Sciences et Techniques de Masuku (USTM), BP 941 Franceville, Gabon; 8 Mongolian Forest Research Association, NGO, #407, 1st Building of Institutes of Mongolian Academy of Sciences, Jukov avenue 77, Bayanzurkh district, Ulaanbaatar, 210351 Mongolia; 9 Center of Forest Ecology and Productivity of the Russian Academy of Sciences, Profsoyuznaya 84/32/14, Moscow, 117997 Russia; 10 Environmental GIS/RS Center, Korea University, Seoul, 02841 Republic of Korea; 11 Department of Harbor and River Engineering, National Taiwan Ocean University, No.2 Pei-Ning Road, Keelung, 20224 Taiwan (R.O.C.); 12 National Science and Technology Center for Disaster Reduction, 9 F., No.200, Sec. 3, Beisin Rd., Xindian District, New Taipei City, 23143 Taiwan (R.O.C.); 13 West University of Timisoara, Bd. Vasile Parvan, no. 4, Timisoara, 300223 Romania; 14 Department of Geography, Gauhati University, Jalukbari, Guwahati, Assam 781014 India; 15 Natural Resources & Agricultural Engineering Department, Faculty of Agriculture, Damanhour University, El-abaadia Campus, Damanhour, 22516 El-behera Egypt; 16 Abhayapuri College, Gauhati University, Abhayapuri, Assam 783384 India; 17 Institute of Meteorology and Climate Research, Atmospheric Environmental Research (IMK-IFU), Karlsruhe Institute of Technology (KIT), Kreuzeckbahnstraße 19, 82467 Garmisch-Partenkirchen, Germany; 18 Department of Meteorology, COMSATS University Islamabad, Islamabad, 45550 Pakistan; 19 Space Applications and Research Complex, Pakistan Space and Upper Atmosphere Research Commission, Islamabad, 44000 Pakistan; 20 Department of Geography, University of Wisconsin-Madison, 550 N. Park Street, Madison, WI 53706 USA; 21 Center for Sustainability and the Global Environment (SAGE), University of Wisconsin‐Madison, 1710 University Avenue, Madison, WI 53726 USA; 22 State Enterprise “Sosnove Forestry”, Shevchenko Str. 125, Sosnove, Berezne district, Rivne region, 34652 Ukraine; 23 Berezne Forestry College of National University of Water and Environmental Engineering, Viacheslava Chornovola str. 23, Bereznе, Rivne region, 34600 Ukraine; 24 Space Research Institute of the Russian Academy of Sciences (IKI), 84/32 Profsoyuznaya Street, Moscow, 117997 Russian Federation; 25 USDA Forest Service, PNW Research Station, Anchorage Forestry Sciences Lab, 161 E 1st Ave., Door 8, Anchorage, AK 99501 USA; 26 Environmental Change Institute, Oxford University Centre for the Environment, University of Oxford, South Parks Road, Oxford, UK

**Keywords:** Environmental impact, Forestry, Biodiversity

## Abstract

Spatially explicit information on forest management at a global scale is critical for understanding the status of forests, for planning sustainable forest management and restoration, and conservation activities. Here, we produce the first reference data set and a prototype of a globally consistent forest management map with high spatial detail on the most prevalent forest management classes such as intact forests, managed forests with natural regeneration, planted forests, plantation forest (rotation up to 15 years), oil palm plantations, and agroforestry. We developed the reference dataset of 226 K unique locations through a series of expert and crowdsourcing campaigns using Geo-Wiki (https://www.geo-wiki.org/). We then combined the reference samples with time series from PROBA-V satellite imagery to create a global wall-to-wall map of forest management at a 100 m resolution for the year 2015, with forest management class accuracies ranging from 58% to 80%. The reference data set and the map present the status of forest ecosystems and can be used for investigating the value of forests for species, ecosystems and their services.

## Abstract (summary)

**Table:** 

Measurement(s)	forest management type
Technology Type(s)	Geo-Wiki toolbox

## Background & Summary

Global knowledge of forest management is critical for informing policies and decision-making on issues such as forest conservation, sustainable forest management, renewable energy, potential supply assessment of forest biomass^1,2^, carbon accounting^3^, and forest restoration practices^4,5^. Having a globally consistent map that characterizes the full range of forest management, from intact forests to plantations and agroforestry, could significantly facilitate these decision-making processes.

Previous work has identified some aspects of forest management in global maps, but this is usually limited to a small number of very broad classes. For example, the intact forest landscapes initiative^6^ provides global maps of intact forests with no signs of human activity and a minimum area of 500 km². This product, however, neglects smaller intact forests that might need attention and protection; it includes sites directly adjacent to clearcuts, which would not be considered intact by our definition; and it omits open forests in the north of Siberia and Canada. The United Nations Environment Programme World Conservation Monitoring Centre provides a natural and modified habitat layer^7^, which has a 1 km resolution and contains four very broad classes: likely modified (14% global forest area), potential modified (11%), potential natural (40%), and likely natural (35%). This data set, however, results from spatial overlay of anthropogenic pressure maps, each with their own uncertainty and definitions. Another quasi-global initiative is the Spatial Database of Planted Trees (SDPT V1.0)^8^. This is a collection of national and local scale maps from various sources, which are not always consistent in their definitions. SDPT V.1.0 recognizes 173 × 10^9^ ha of planted forest (59% of what the Food and Agriculture Organization’s Global Forest Resources Assessment (FAO FRA) reported) and 50 × 10^9^ ha of tree crops. Schulze *et al*.^9^ have downscaled the FAO FRA country statistics to create a global map at a 1 km resolution. The authors used 21 socio-economic and bio-physical predictor variables, but only 789 training points, collected from the literature without stratification or randomization, which limits the ability to capture spatial variability accurately.

Hence, to date, a global map of forest management covering the full range of classes has not yet been produced. Remote sensing (RS) and RS-based products have been widely used in the aforementioned studies, but, in terms of forest management, most of them identify only areas with intensive tree management (e.g., short rotation plantations, oil palm plantations^10^). This can be partly explained by the fact that there are insufficient reference data available, and the time series from RS are too short to cover typical forest management rotation cycles.

Here, we present the first global reference data set of forest management and a first prototype of a global forest management map derived from RS. Forests and forestry are very diverse globally, so we generalized the forest management practices that occur across countries into a set of consistent definitions, including (i) intact forests; (ii) forests with signs of human impact, including logging; (iii) planted forests; (iv) plantation forests with a rotation period of up to 15 years; (v) oil palm plantations; and (vi) agroforestry. We use the term “forest management” throughout the paper but note that our classification also contains other land-use classes with trees, such as agroforestry and oil palm plantations.

**Fig. 1:**
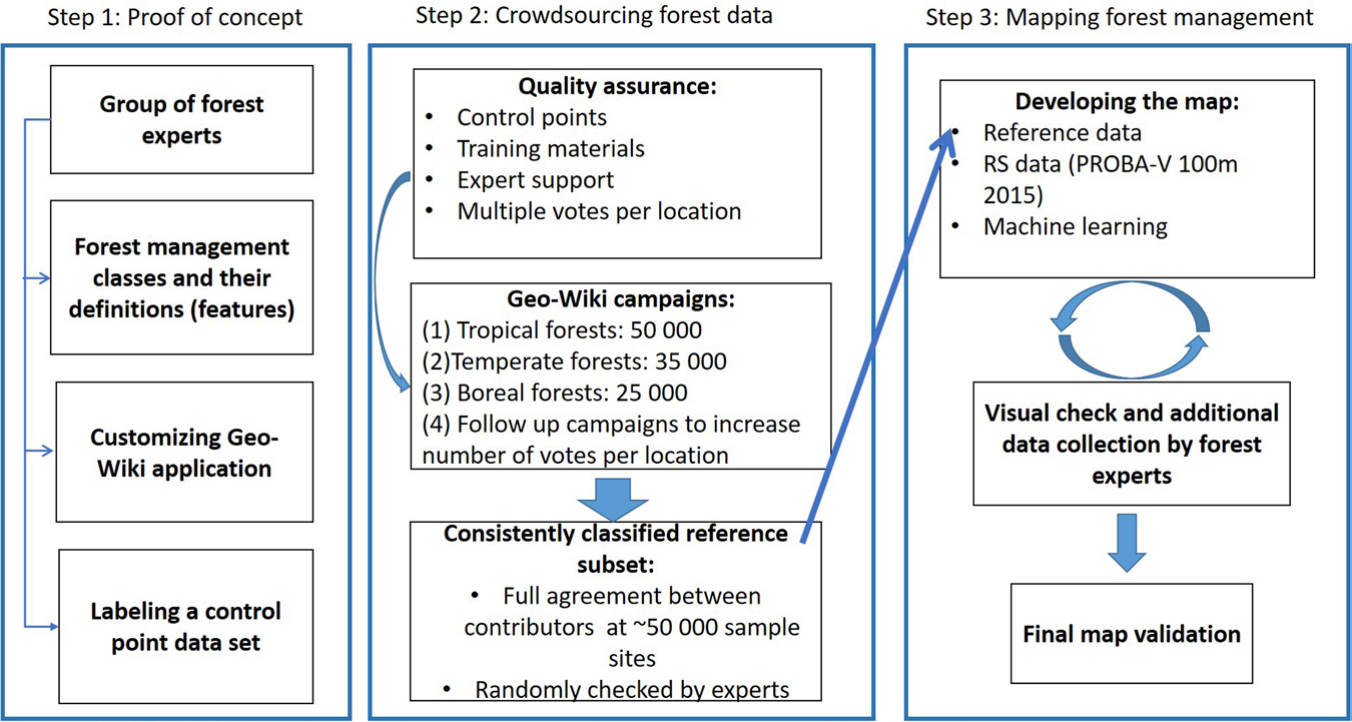
Study design.

To build the reference data set, we invited experts from different parts of the world. Together, we designed and implemented a platform with an appropriate interface (see https://www.geo-wiki.org/) for crowdsourcing campaigns in which the forest experts and Geo-Wiki participants labelled time series of very high-resolution imagery to collect reference data at 226,322 locations globally. Using these data, we developed a wall-to-wall global forest management map at a 100 m resolution (PROBA-V geometry) for the year 2015. To minimize the amount of resources needed for mapping, we used the already existing classification workflow that produced the Copernicus Land Cover product for 2015^11^. Figure 1 presents the study design.

The reference data set is of sufficient quantity and quality to be used at regional and local scales for carrying out various forest related research. The forest management map is a prototype of generalised forest management classes and could be used in various applications, either at global, continental, or large regional scales.

## Methods

### Reference data collection

In February 2019, we involved forest experts from different regions around the world and organized a workshop to (1) discuss the variety of forest management practices that take place in various parts of the world; (2) explore what types of forest management information could be collected by visual interpretation of very high-resolution images from Google Maps and Microsoft Bing Maps, in combination with Sentinel time series and Normalized Difference Vegetation Index (NDVI) profiles derived from Google Earth Engine (GEE); (3) generalize and harmonize the definitions at global scale; (4) finalize the Geo-Wiki interface for the crowdsourcing campaigns; and (5) build a data set of control points (or the expert data set), which we used later to monitor the quality of the crowdsourced contributions by the participants. Based on the results of this analysis, we launched the crowdsourcing campaigns by involving a broader group of participants, which included people recruited from remote sensing, geography and forest research institutes and universities. After the crowdsourcing campaigns, we collected additional data with the help of experts. Hence, the final reference data consists of two parts: (1) a randomly stratified sample collected by crowdsourcing (49,982 locations); (2) a targeted sample collected by experts (176,340 locations, at those locations where the information collected from the crowdsourcing campaign was not large enough to ensure a robust classification).

### Definitions

Table 1 contains the initial classification used for visual interpretation of the reference samples and the aggregated classes presented in the final reference data set. For the Geo-Wiki campaigns, we attempted to collect information (1) related to forest management practices and (2) recognizable from very high-resolution satellite imagery or time series of vegetation indices. The final reference data set and the final map contain an aggregation of classes, i.e., only those that were reliably distinguishable from visual interpretation of satellite imagery.

**Table 1:** Forest management classes and definitions.

Map ID	Final aggregated classes	Classification used in the Geo-Wiki campaigns
11	Naturally regenerating forests without any signs of management, including primary forests	Forests with no or very low human impact: • “not disturbed” – natural forest without detectable evidence of any disturbances within the 100 m pixel and within 500 m in any direction. • “with human impact nearby” – forest in the classified 100 m pixel is not disturbed, but there are roads or evidence of non-forest management related human activities (e.g., houses, small agricultural fields) situated nearby (within 500 m in any direction). • “degraded or disturbed” – no human activities in the 100 m pixel or nearby. Forest has been disturbed by natural disturbances, i.e., wildfire, wind throw, flooding, or insect/disease outbreaks.
20	Naturally regenerating forests with signs of forest management, e.g., logging, clear cuts etc.	Forests with signs of management/cuts in the 100 m pixel or nearby including: • “naturally regenerated forests” – forest is managed with signs of logging (including selected logging) in the 100 m pixel or nearby, but there are no signs of planting. This also includes semi-natural forests – forests without major forest management interventions, which are very similar visually to naturally regenerating forests.
31	Planted forests (rotation >15 years)	• “planted forest” – forest is managed and there are signs that the forest has been planted in the 100 m pixel. Rotation time is relatively long (>15 years).
32	Plantation forest (rotation ≤15 years)	Plantation forests: • Intensively managed forest plantations for timber with short rotation (15 years max).
40	Oil palm plantations	• “oil palm” – palms have very distinguishable crown shapes.
53	Agroforestry	Other landscapes: • “fruit trees (olives, apples, nuts, cocoa, etc.)”. • “Tree shelter belts, small forest patches” – group of trees on cropland/pastures in lines or patches. • “Agroforestry or sparse trees on agricultural fields” – mixed crops (including trees) or individual trees on cropland or pasture. • “Shifting cultivation” – a form of agriculture, in which an area is cleared of vegetation and cultivated for a few years and then abandoned for a new area until its fertility has been naturally restored. Usually, one can see pieces of land with all the stages of this process. • “trees in urban/built-up areas” – buildings or infrastructure dominant in the 100 m pixel or surroundings.

### Sampling design for the crowdsourcing campaigns

**Fig. 2:**
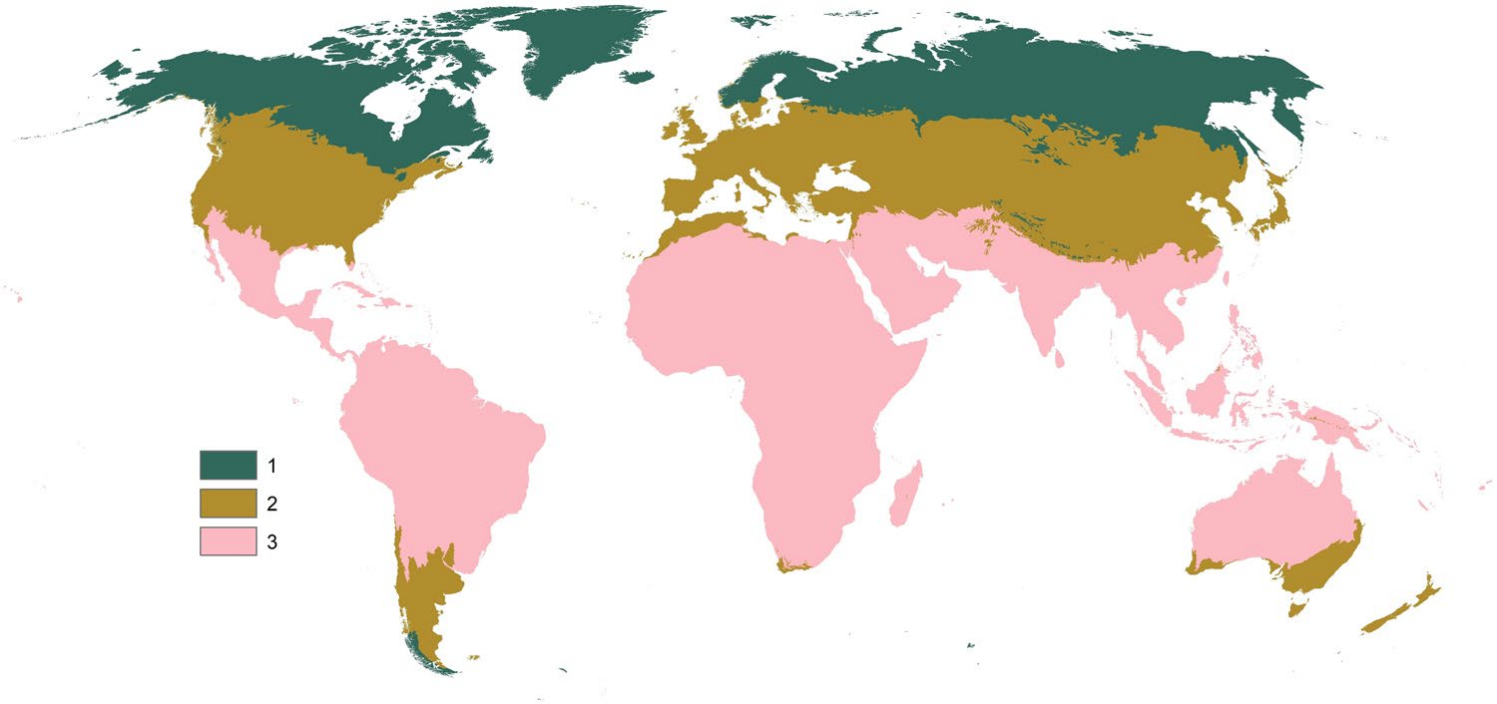
Biomes for sampling stratification (1 – boreal, 2 – temperate, 3 – sub-tropical and tropical).

Initially, we generated a random stratified sample of 110,000 sites globally. The total number of sample sites was chosen based on experiences from past Geo-Wiki campaigns^12^, a practical estimation of the potential number of volunteer participants that we could engage in the campaign, and the expected spatial variation in forest management. We used two spatial data sets for the stratification of the sample: World Wildlife Fund (WWF) Terrestrial Ecoregions^13^ and Global Forest Change^14^. The samples were stratified into three biomes, based on WWF Terrestrial Ecoregions (Fig. 2): boreal (25 000 sample sites), temperate (35,000 sample sites) and tropical (50,000 sample sites). Within each biome, we used Hansen’s^14^ Global Forest Change maps to derive areas with “forest remaining forest” 2000–2015, “forest loss or gain”, and “permanent non-forest” areas.

The sample size was determined from previous experiences, taking into account the expected spatial variation in forest management within each biome. Tropical forests had the largest sample size because of increasing commodity-driven deforestation^15^, the wide spatial extent of plantations, and slash and burn agriculture. Temperate forests had a larger sample compared to boreal forests due to their higher fragmentation. Each sample site was classified by at least three different participants, thus accounting for human error and varying expertise^16–18^. At a later stage, following a preliminary analysis of the data collected, we increased the number of sample sites to meet certain accuracy thresholds for every mapped class (aiming to exceed 75% accuracy).

### The Geo‐Wiki application

**Fig. 3:**
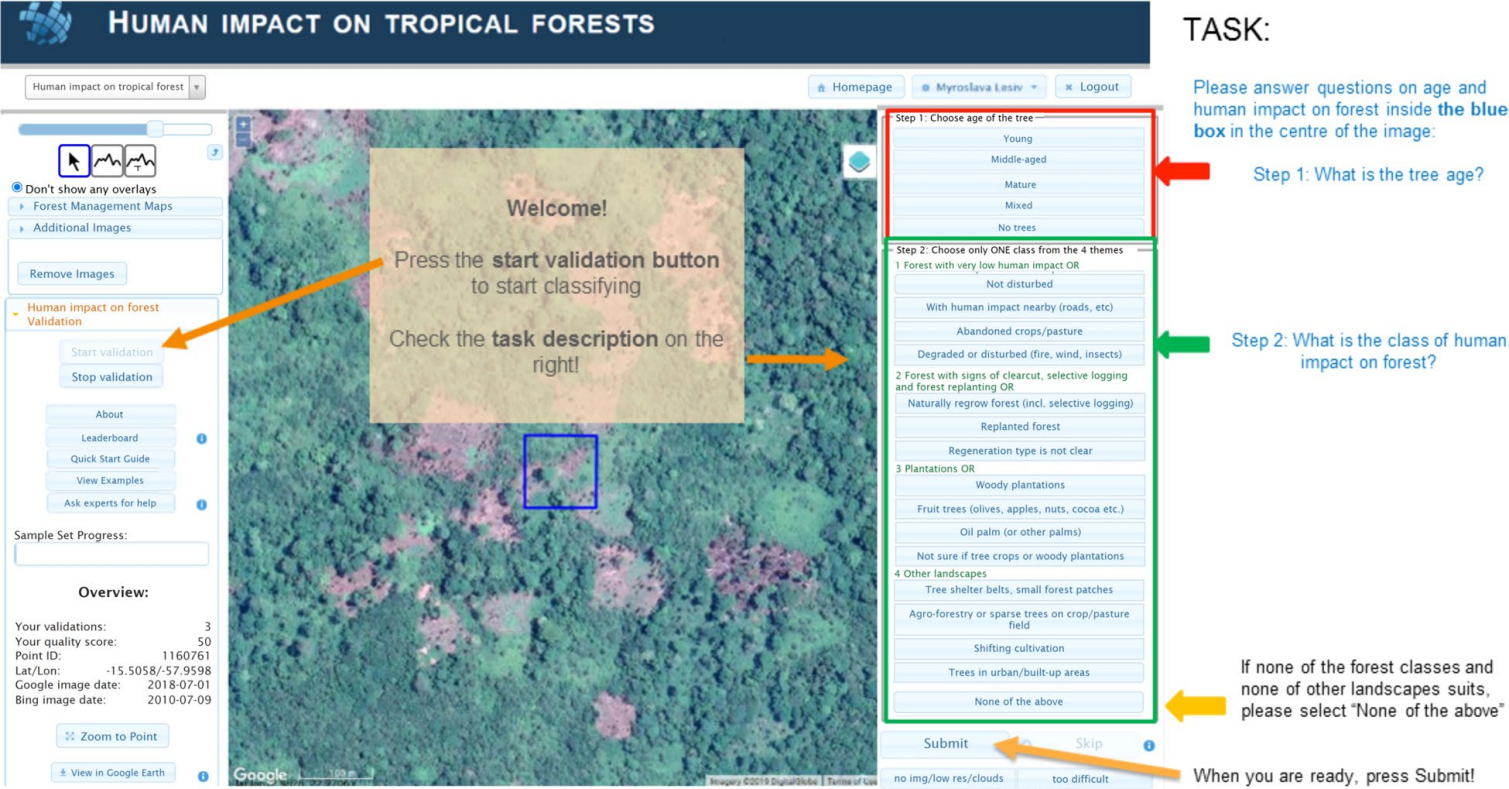
Screenshot of the Geo‐Wiki interface showing a very high-resolution image from Google Maps and a sample site as a 100 mx100 m blue square, which the participants classified based on the forest management classes on the right.

Geo‐Wiki.org is an online application for crowdsourcing and expert visual interpretation of satellite imagery, e.g., to classify land cover and land use. This application has been used in several data collection campaigns over the last decade^16,19–23^. Here, we implemented a new custom branch of Geo‐Wiki (‘Human impact on Forest’), which is devoted to the collection of forest management data (Fig. 3). Various map overlays (including satellite images from Google Maps, Microsoft Bing Maps and Sentinel 2), campaign statistics and tools to aid interpretation, such as time series profiles of NDVI, were provided as part of this Geo‐Wiki branch, giving users a range of options and choices to facilitate image classification and general data collection. Google Maps and Microsoft Bing Maps include mosaics of very high-resolution satellite and aerial imagery from different time periods and multiple image providers, including the Landsat satellites operated by NASA and USGS as base imagery to commercial image providers such as Digital Globe. More information on the spatial and temporal distribution of very high-resolution satellite imagery can be found in Lesiv *et al*.^24^. This collection of images was supplied as guidance for visual interpretation^16,20^. Participants could analyze time series profiles of NDVI from Landsat, Sentinel 2 and MODIS images, which were derived from Google Earth Engine (GEE). More information on tools can be found in Supplementary file 1.

The blue box in Fig. 3 corresponds to 100 m × 100 m pixels aligned with the Sentinel grid in UTM projection. It is the same geometry required for the classification workflow that is used to produce the Copernicus Land Cover product for 2015^11^.

Before starting the campaign, the participants were shown a series of slides designed to help them gain familiarity with the interface and to train them in how to visually determine and select the most appropriate type of land use and forest management classes at each given location, thereby increasing both consistency and accuracy of the labelling tasks among experts. Once completed, the participants were shown random locations (from the random stratified sample) on the Geo‐Wiki interface and were then asked to select one of the forest management classes outlined in the Definition section (see Table 1 above).

Alternatively, if there was either insufficient quality in the available imagery, or if a participant was unable to determine the forest management type, they could skip such a site (Fig. 3). If a participant skipped a sample site because it was too difficult, other participants would then receive this sample site for classification, whereas in the case of the absence of high-resolution satellite imagery, i.e., Google Maps and Microsoft Bing Maps, this sample site was then removed from the pool of available sample sites. The skipped locations were less than 1% of the total amount of locations assigned for labeling. Table 2 shows the distribution of the skipped locations by countries, based on the subset of the crowdsourced data where all the participants agreed.

**Table 2:** Distribution of the skipped locations by countries.

Countries with more than 10 samples	Number of samples	Percentage in the subset of the crowdsourced data set where all the participants agreed, %
Indonesia	70	4
Brazil	48	1
Colombia	24	8
Russia	28	<1
Canada	22	3
Malaysia	18	3
Gabon	18	28
Venezuela	16	4
Guyana	13	23
Peru	12	5
Global	356	<1

### Quality assurance and data aggregation of the crowdsourced data

Based on the experience gained from previous crowdsourcing campaigns^12,19^, we invested in the training of the participants (130 persons in total) and overall quality assurance. Specifically, we provided initial guidelines for the participants in the form of a video and a presentation that were shown before the participants could start classifying in the forest management branch (Supplementary file 1). Additionally, the participants were asked to classify 20 training samples before contributing to the campaign. For each of these training samples, they received text‐based feedback regarding how each location should be classified. Summary information about the participants who filled in the survey at the end of the campaign (i.e., gender, age, level of education, and their country of residence) is provided in the Supplementary file 2. We would like to note that 130 participants is a high number, especially taking the complexity of the task into consideration.

Furthermore, during the campaign, sample sites that were part of the “control” data set were randomly shown to the participants. The participants received text-based feedback regarding whether the classification had been made correctly or not, with additional information and guidance. By providing immediate feedback, our intention was that participants would learn from their mistakes, increasing the quality and classification accuracy over time. If the text‐based feedback was not sufficient to provide an understanding of the correct classification, the participants were able to submit a request (“Ask the expert”) for a more detailed explanation by email.

The control set was independent of the main sample, and it was created using the same random stratified sampling procedure within each biome and the stratification by Global Forest Change maps^14^ (see “Sample design” section). To determine the size of the control sample, we considered two aspects: (a) the maximum number of sample sites that one person could classify during the entire campaign; (b) the frequency at which control sites would appear among the task sites (defined at 15%, which is a compromise between the classification of as many unknown locations as possible and a sufficient level of quality control, based on previous experience). Our control sample consisted of 5,000 sites. Each control sample site was classified twice by two different experts. When the two experts agreed, these sample sites were added to the final control sample. Where disagreement occurred (in 25% of cases), these sample sites were checked again by the experts and revised accordingly. During the campaign, participants had the option to disagree with the classification of the control site and submit a request with their opinion and arguments. They received an additional quality score in the situation when they were correct, but the experts were not. This procedure also ensured an increase in the quality of the control data set.

To incentivize participation and high-quality classifications, we offered prizes as part of the campaign design. The ranking system for the prize competition considered both the quality of the classifications and the number of classifications provided by a participant. The quality measure was based on the control sample discussed above. The participants randomly received a control point, which was classified in advance by the experts. For every control point, a participant could receive a maximum of +30 points (fully correct classification) to a minimum of −30 points (incorrect classification). In the case where the answer was partly correct (e.g., the participant correctly classified that the forest is managed, but misclassified the regeneration type), they received points ranging from 5 to 25.

The relative quality score for each participant was then calculated as the total sum of gained points divided by the maximum sum of points that this participant could have earned. For any subsequent data analysis, we excluded classifications from those participants whose relative quality score was less than 70%. This threshold corresponds to an average score of 10 points at each location (out of a maximum of 30 points), i.e., where participants were good at defining the aggregated forest management type but may have been less good at providing the more detailed classification.

Unfortunately, we observed some imbalance in the proportion of participants coming from different countries, e.g. there were not so many participants from the tropics. This could have resulted in interpretation errors, even when all the participants agreed on a classification. To address this, we did an additional quality check. We selected only those sample sites where all the participants agreed and then randomly checked 100 sample sites from each class. Table 3 summarizes the results of this check and explains the selection of the final classes presented in Table 1.

**Table 3:** Qualitative analysis of the reference sample sites with full agreement.

Biome	Summary of the analysis	Actions taken
Tropical forests	• Locations with no images and “no forest” (<5% of tree canopy) – no issues detected. • “Forest with no or very low human impact” – we found 2% of locations with signs of human activities nearby and 1% of degraded forests. • “Forest with signs of human activities nearby” – no issues detected. • “Naturally regenerating forest” – no issues detected. • “Plantation forests” – no issues detected. • “Fruit trees (olives, apples, nuts, cocoa, etc.)” were sometimes confused with young oil palm plantations, which is a separate class in our legend. • “Oil palm” plantations – no issues detected. • “Tree shelter belts, small forest patches” were sometimes confused with naturally regenerating forests and with agroforestry. • “Agroforestry or sparse trees on agricultural fields” were sometimes confused with fruit plantations. • “Trees in urban/built-up areas” were confused with fruit plantations and “Agroforestry or sparse trees on agricultural fields”, and “naturally regenerating forests”. • There were many mixed pixels with fruit trees plantations, agroforestry, tree shelterbelts and small forest patches.	• All locations with the following classes were revised by experts: “fruit trees (olives, apples, nuts, cocoa, etc.)”, “tree shelter belts and small forest patches”, “agroforestry or sparse trees on agricultural fields”, and “Trees in urban/built-up areas”; • Merged “forest with no or very low human impact”, “forest with human impact nearby”, and “degraded and disturbed” forests into one class called “Naturally regenerating forest without any signs of management”. • Merged “fruit trees (olives, apples, nuts, cocoa, etc.)”, “tree shelter belts, small forest patches”, “Agroforestry or sparse trees on agricultural fields”, and “Trees in urban/built-up areas” into one class “Agroforestry”.
Temperate forests	• Locations with no images – no issues detected. • Locations with “no forest” – we found only a few misclassifications, which included a poplar plantation that was not visible on the Microsoft Bing Maps image, degraded forest dominated by snags, and abandoned fields that are reverting to forests. • “Forest with no or very low human impact” and “forests with human impact nearby” were correctly classified, with the exception of shrubland in Australia, which was partly misclassified as forest. “Naturally regenerating forest” had only a few misclassifications such as “planted forests” mapped as “naturally regenerating forest”. • “Planted forest” were correctly classified with the exception of planted forests in the USA and China that were confused with “naturally regenerating forests”. • “Plantation forests” – no issues detected. • “Fruit trees (olives, apples, nuts, cocoa, etc.)” were sometimes confused with pine nut plantations. • “Tree shelter belts, small forest patches” had mistakes related to belts between young plantations or naturally regenerating forests. • “Agroforestry or sparse trees on agricultural fields” – this category was not understood very well. Many misclassified points were either sparse natural forests or naturally regenerating forests take place.	• Revisited and replaced if necessary locations classified as being “no forest”, with “forest with no or very low human impact” in Australia, revisited and replaced if necessary “naturally regenerating forest” with “planted forest” in the USA and China, revisited and replaced if necessary “fruit trees (olives, apples, nuts, cocoa, etc.)” with “Agroforestry or sparse trees on agricultural fields”. • Classes were merged similarly to the tropic’s category, to ensure global map consistency.
Boreal forests	• Locations with no image available are classified correctly. • “No forest” – no issues detected. • “Forest with no or very low human impact” confused with degraded forest. • “Forest with human impact nearby” confused with “naturally regenerating forests”. • “Naturally regenerating forest” confused with “planted forest” in Sweden and Finland. • “Planted forest” were correct, except for Moscow region, Russia. • No issues detected with agroforestry, tree shelterbelts and trees in urban areas.	• Revisited and replaced, if necessary, “forest with human impact nearby” with “naturally regenerating forests” in Finland and Sweden, and planted forests around Moscow, Russia. • Classes were merged similarly to the tropics, to ensure global map consistency.

As a result of the actions outlined in Table 3, we compiled the final reference data set, which consisted of 49,982 consistent sample sites.

### Additional expert data collection

**Fig. 4:**
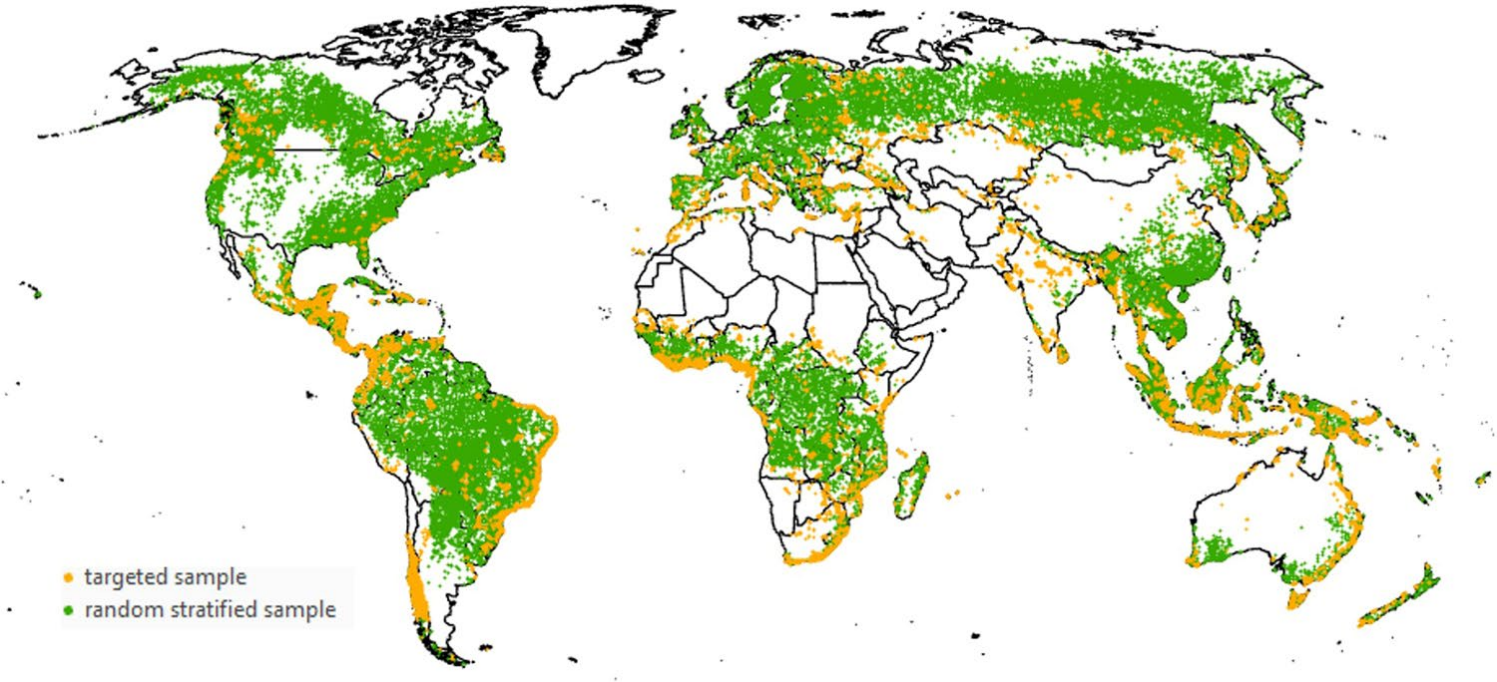
Distribution of reference locations.

We used the reference data set to produce a test map of forest management (the classification algorithm used is described in the next section). By checking visually and comparing against the control data set, we found that the map was of insufficient quality for many locations, especially in the case of heterogeneous landscapes. While several reasons for such an unsatisfactory result are possible, the experts agreed that a larger sample size would likely increase the accuracy of the final map, especially in areas of high heterogeneity and for forest management classes that only cover a small spatial extent. To increase the amount of high-quality training data and hence to improve the map, we collected additional data using a targeted approach. In practice, the map was uploaded to Geo-Wiki, and using the embedded drawing tools, the experts randomly checked locations on the map, focusing on their region of expertise and added classified polygons in locations where the forest management was misclassified. To limit model overfitting and oversampling of certain classes, the experts also added points for correctly mapped classes to keep the density of the points the same. This process involved a few iterations of collecting additional points and training the classification algorithm until the map accuracy reached 75%. In total, we collected an additional 176,340 training points. With the 49,982 consistent training points from the Geo-Wiki campaigns, this resulted in 226,322 (Fig. 4). This two-pronged approach would not have been possible without the exhaustive knowledge obtained from running the initial Geo-Wiki campaigns, including numerous questions raised by the campaign participants. Figure 4 also highlights in yellow the areas of very high sampling density, I.e., those collected by the experts. The sampling intensity of these areas is much higher in comparison with the randomly distributed crowdsourced locations, and these are mainly areas with very mixed forest classes or small patches, in most cases, including plantations.

### Classification algorithm

**Fig. 5:**
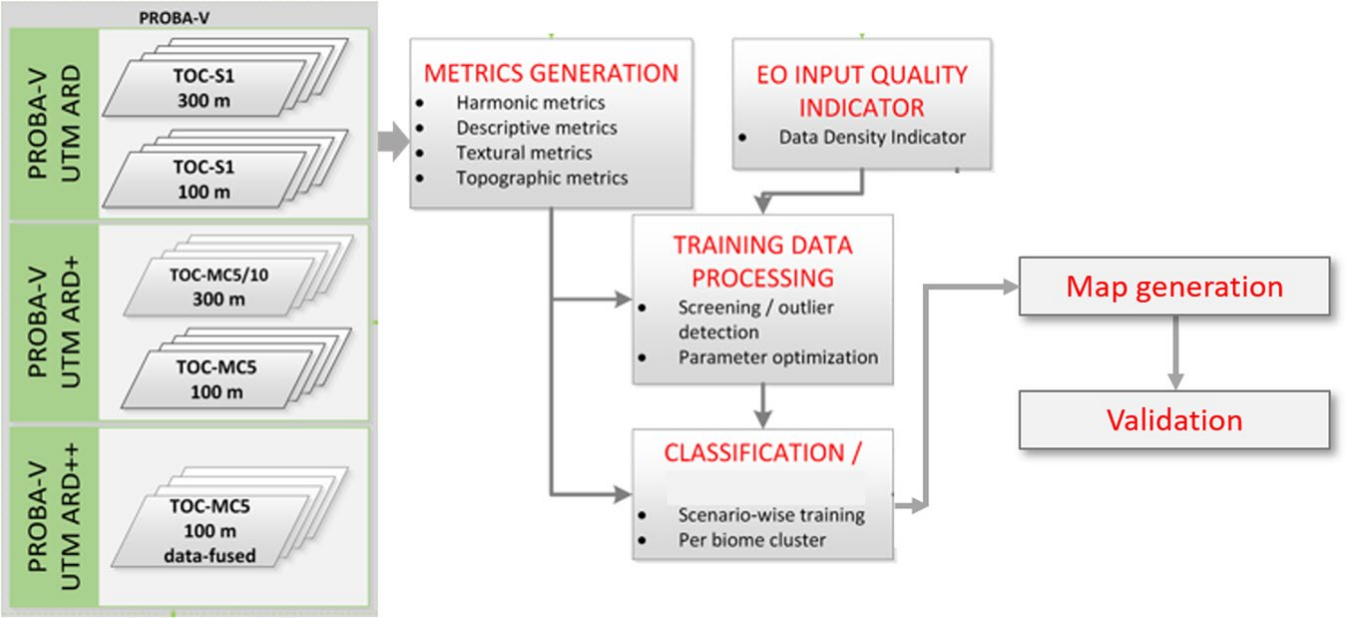
Workflow overview for the generation of the Copernicus Global Land Cover Layers. Adapted from the Algorithm Theoretical Basis Document^25^.

To produce the forest management map for the year 2015, we applied a workflow that was developed as part of the production of the Copernicus Global Land Services land cover at 100 m resolution (CGLS-LC100) collection 2 product^11^. A brief description of the workflow (Fig. 5), focusing on the implemented changes, is given below. A more thorough explanation, including detailed technical descriptions of the algorithms, the ancillary data used, and the intermediate products generated, can be found in the Algorithm Theoretical Basis Document (ATBD) of the CGLS-LC100 collection 2 product^25^.

The CGLS-LC100 collection 2 processing workflow can be applied to any satellite data, as it is unspecific to different sensors or resolutions. While the CGLS-LC100 Collection 2 product is based on PROBA-V sensor data, the workflow has already been tested with Sentinel 2 and Landsat data, thereby using it for regional/continental land cover (LC) mapping applications^11,26^. For generating the forest management layer, the main Earth Observation (EO) input was the PROBA-V UTM Analysis Ready Data (ARD) archive based on the complete PROBA-V L1C archive from 2014 to 2016. The ARD pre-processing included geometric transformation into a UTM coordinate system, which reduced distortions in high northern latitudes, as well as improved atmospheric correction, which converted the Top-of-Atmosphere reflectance to surface reflectance (Top-of-Canopy). In a further processing step, gaps in the 5-daily PROBA-V UTM multi-spectral image data with a Ground Sampling Distance (GSD) of ~0.001 degrees (~100 m) were filled using the PROBA-V UTM daily multi-spectral image data with a GSD of ~0.003 degrees (~300 m). This data fusion is based on a Kalman filtering approach, as in Sedano *et al*.^27^, but was further adapted to heterogonous surfaces^25^. Outputs from the EO pre-processing were temporally cleaned by using the internal quality flags of the PROBA-V UTM L3 data, a temporal cloud and outlier filter built on a Fourier transformation. This was done to produce consistent and dense 5-daily image stacks for all global land masses at 100 m resolution and a quality indicator, called the Data Density Indicator (DDI), used in the supervised learning process of the algorithm.

Since the total time series stack for the epoch 2015 (a three-year period including the reference year 2015 +/− 1 year) would be composed of too many proxies for supervised learning, the time and spectral dimension of the data stack had to be condensed. The spectral domain was condensed by using Vegetation Indices (VIs) instead of the original reflectance values. Overall, ten VIs based on the four PROBA-V reflectance bands were generated, which included: Normalized Difference Vegetation Index (NDVI); Enhanced Vegetation Index (EVI); Structure Intensive Pigment Index (SIPI); Normalized Difference Moisture Index (NDMI); Near-Infrared reflectance of vegetation (NIRv); Angle at NIR; HUE and VALUE of the Hue Saturation Value (HSV) color system transformation. The temporal domain of the time series VI stacks was then condensed by extracting metrics, which are used as general descriptors to enable distinguishing between the different LC classes. Overall, we extracted 266 temporal, descriptive, and textual metrics from the VI times series stacks. The temporal descriptors were derived through a harmonic model, fitted through the time series of each of the VIs based on a Fourier transformation^28,29^. In addition to the seven parameters of the harmonic model that describe the overall level and seasonality of the VI time series, 11 descriptive statistics (mean, standard deviation, minimum, maximum, sum, median, 10th percentile, 90th percentile, 10th – 90th percentile range, time step of the first minimum appearance, and time step of the first maximum appearance) and one textural metric (median variation of the center pixel to median of the neighbours) were generated for each VI. Additionally, the elevation, slope, aspect, and purity derived at 100 m from a Digital Elevation Model (DEM) were added. Overall, 270 metrics were extracted from the PROBA-V UTM 2015 epoch.

The main difference to the original CGLS-LC100 collection 2 algorithms is the use of forest management training data instead of the global LC reference data set, as well as only using the discrete classification branch of the algorithm. The dedicated regressor branch of the CGLS-LC100 collection 2 algorithm, i.e., outputting cover fraction maps for all LC classes, was not needed for generating the forest management layer.

In order to adapt the classification algorithm to sub-continental and continental patterns, the classification of the data was carried out per biome cluster, with the 73 biome clusters defined by the combination of several global ecological layers, which include the ecoregions 2017 dataset^30^, the Geiger-Koeppen dataset^31^, the global FAO eco-regions dataset^32^, a global tree-line layer^33^, the Sentinel-2 tiling grid and the PROBA-V imaging extent;^30,31^ this, effectively, resulted in the creation of 73 classification models, each with its non-overlapping geographic extent and its own training dataset. Next, in preparation for the classification procedure, the metrics of all training points were analyzed for outliers, as well as screened via an all-relevant feature selection approach for the best metric combinations (i.e., best band selection) for each biome cluster in order to reduce redundancy between parameters used in the classification. The best metrics are defined as those that have the highest separability compared to other metrics. For each metric, the separability is calculated by comparing the metric values of one class to the metric values of another class; more details can be found in the ATBD^25^. The optimized training data set, together with the quality indicator of the input data (DDI data set) as a weight factor, were used in the training of the Random Forest classifier. Moreover, a 5-fold cross-validation was used to optimize the classifier parameters for each generated model (one per biome).

**Fig. 6:**
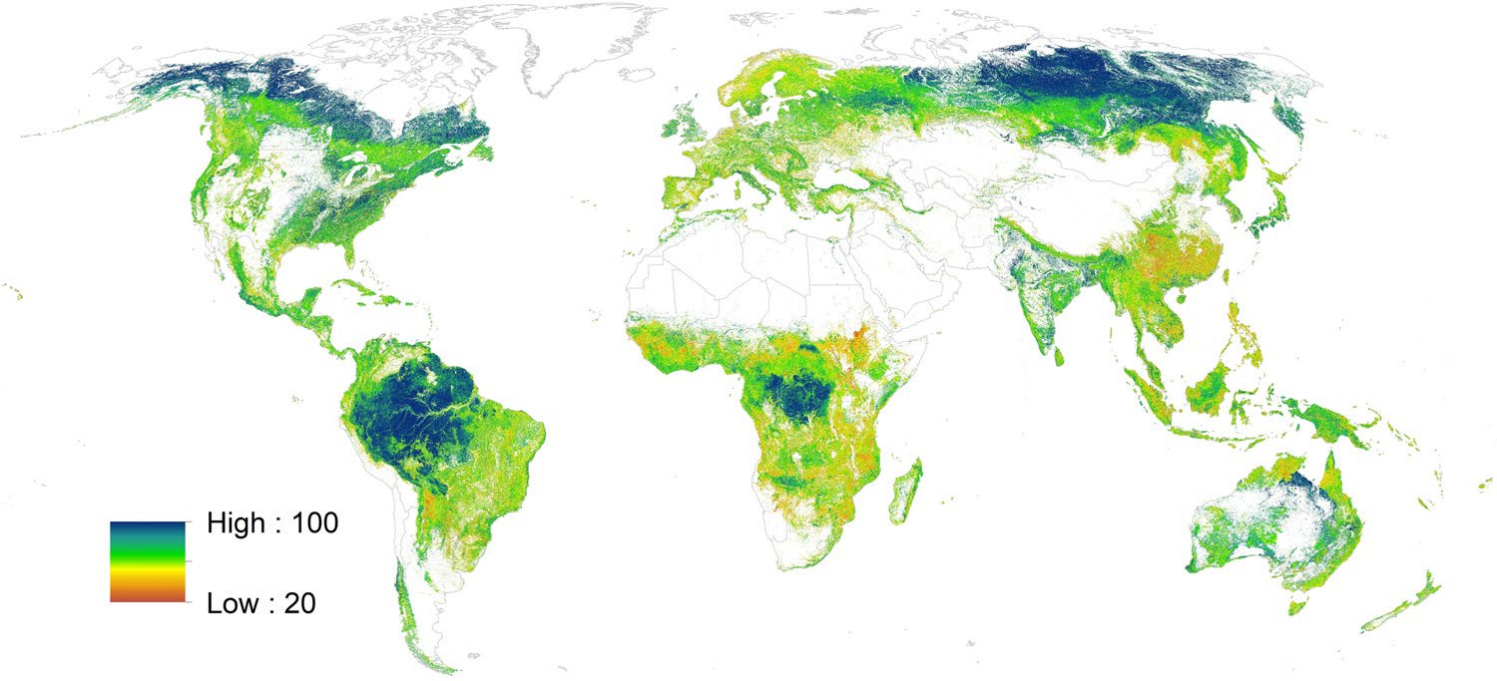
The predicted class probability by the Random Forest classification.

Finally, the Random Forest classification was used to produce a hard classification, showing the discrete class for each pixel, as well as the predicted class probability. In the last step, the discrete classification results (now called the forest management map) are modified by the CGLS-LC100 collection 2 tree cover fraction layer^29^. Therefore, the tree cover fraction layer, showing the relative distribution of trees within one pixel, was used to remove areas with less than 10% tree cover fraction in the forest management layer, following the FAO definition of forest. Figure 6 shows the class probability layer that illustrates the model behavior, highlighting the areas of class confusion. This layer shows that there is high confusion between forest management classes in heterogeneous landscapes, e.g., in Europe and the Tropics while homogenous landscapes, such as Boreal forests, are mapped with high confidence. It is important to note that a low probability does not mean that the classification is wrong.

## Data Records

We provide six data records^34^:

1. The reference data set as a comma-separated file (.csv) with the following attributes:
  “ID” is a unique location identifier.
  “Latitude, Longitude” are centroid coordinates of a 100 m × 100 m pixel.
  “Land_use_ID “is a land use (forest management) class:
    0 – not forest;
    11 – Naturally regenerating forest without any signs of management, including primary forests;
    20 – Naturally regenerating forest with signs of management, e.g., logging, clear cuts etc;
    31 – Planted forests;
    32 –Plantation forests (rotation time up to 15 years);
    40 – Oil palm plantations;
    53 – Agroforestry;
    −1 – samples marked as unsure in the control data set;
    1 – samples with very high resolution imagery unavailable in the control data set.
  “Flag” identifies a data origin: 1 – the crowdsourced locations (random stratified sample), 2 – the control data set (random stratified), 0 – the additional experts’ classifications following the opportunistic approach (non-random).
2. The 100 m forest management map in a geoTiff format (see Fig. 7) with the classes presented (1).

**Fig. 7:**
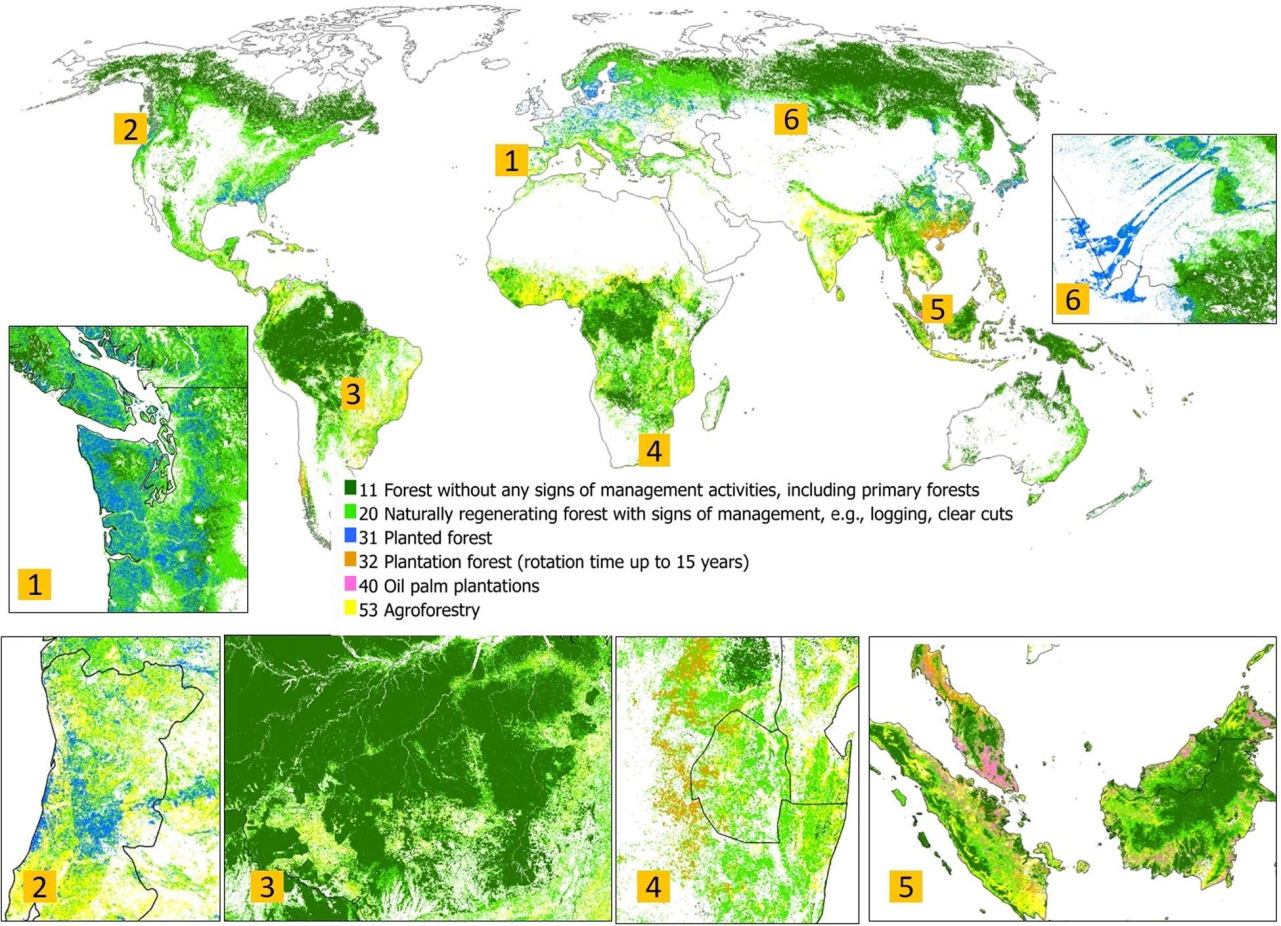
Forest management map with the following classes: 11 – Naturally regenerating forest without any signs of management, including primary forests, 20 – Naturally regenerating forest with signs of management, e.g., logging, clear cuts, 31– Planted forest; 32 – Plantation forests (rotation time up to 15 years), 40 – Oil palm plantations, 53 – Agroforestry. Six areas distributed across different continents are provided with more detailed insets: (1) planted forests in Portugal; (2) planted forests in Washington state, the USA and Vancouver Island; (3) Brazil; (4) Plantation forests in Eswatini, South Africa; (5) Peninsular Malaysia, Borneo and Sumatra; (6) planted forest in Russia and Kazakhstan.
3. The predicted class probability from the Random Forest classification in a geoTiff format (see Fig. 6).
4. Validation data set as a comma-separated file (.csv) with the following attributes:
  “ID” is a unique location identifier;
  “pixel_center_x”, “pixel_center_y” are centroid coordinates of a 100 m × 100 m pixel in lat/lon projection;
  “first_landuse_class” is a land use class, as in (1);
  “second_landuse_class” is a second possible land use class, as in (1), identified in case it was difficult to assign one class with high confidence.
5. Original crowdsourced data set as a.csv table.
6. Compiled FAO FRA forest statistics and mapped classes by countries into one table (.csv format).

## Technical Validation

### Reference data set

We have explained the quality assurance measures in detail in the section “Quality assurance and data aggregation”. In short, the data set provided is based on forest expert knowledge. We excluded all locations with high uncertainty based on expert feedback; therefore, we consider the reference data set to be of the highest quality for validation purposes.

### Forest management map

We were unable to use the control data set for map validation because it does not follow a suitable sample design for rigorous statistical validation^35^. Therefore, we carried out an independent validation of the map by following the procedure set out in Olofsson *et al*.^36^, which allows the confidence intervals to be estimated and the area estimates to be adjusted based on the confusion matrix. We applied a random stratified sampling design to validate the map at the global level using the mapped classes as strata. We estimated that to calculate the overall accuracy of the map with a targeted accuracy of 75%, we would need approximately 2100 locations. Once we generated the sample, it was interpreted by the same set of forest experts who classified the control sample and harmonized the definitions, using Geo-Wiki. The experts were asked to identify one of the classes from Table 1. According to the guidelines^35^, the uncertain classifications should not be deleted. Hence, if it was difficult to determine a unique class, the experts were asked to suggest the second most likely classification at each location with uncertainty. These second classifications were used in the accuracy assessment. For example, if a validation site was classified as “planted forest” as the first classification and “naturally regenerating” as the second classification, and the mapped class was “planted forest”, then a value of 0.5 was added to the cell of the confusion matrix in the row and column “planted forest” while the other 0.5 was added to the cell in the row and column “naturally regenerating forest”.

We found the overall accuracy of the map to be 82 ± 0.01%. Table 4 provides the confusion matrix with per class accuracies. Out of 2100 locations, we had images for 2072. Hence, we consider the remaining 28 locations (~2%) as a bias of our estimates.

**Table 4:** Confusion matrix.

Mapped class id	Reference class							User’s, %	CI*, %	Produ–cer’s, %	CI*, %
	0	11	20	31	32	40	53				
0	429	9	13	1.5	1	0	18.5	91	2.6	94	1.1
11	21	281	45	2	1	0	2	80	4.2	85	4.1
20	28.5	36	246.5	39	8.5	4.5	23	64	4.8	65	5
31	8.5	3	38.5	153.5	2	0	10.5	71	6.1	35	7
32	13	4	44.5	13.5	129	1	17	58	6.5	36	13.6
40	6.5	2	22.5	0	6	108	9	70	7.3	24	12.7
53	34	6.5	46	3.5	4	4.5	171.5	64	5.8	49	8.2
Overall accuracy								83	1.9		

*CI – confidence interval.

From the confusion matrix, it follows that planted forests (class 31) are, unsurprisingly, underestimated. This class is confused with naturally regenerated managed forests (20). The confusion matrix also indicates that there should be double the amount of planted forests than is currently shown on the map. Classes such as agroforestry, plantation forests and oil palm plantations are also underestimated due to confusion with class 20. This confusion is observed in tropical countries and could be explained by using only optical RS data, which has limited number of valid observations due to cloud coverage. Naturally regenerated forests without any signs of management (class 11) are mapped with a rather high user accuracy of 80%. This may be explained by the fact that these are quite remote homogenous areas that are easy to map from a RS point of view.

We also calculated the mapped areas of each class and the adjusted areas considering commission and omission errors as presented in Table 5. Please note that the calculations below may include biases related to the interpretation errors, which we assume are rather small.

**Table 5:** Total area by forest management class.

Mapped class id	Mapped class name	Mapped area, 10^6^ ha	Adjusted area, 10^6^ ha	CI*, 10^6^ ha
0	No forest	7757	7463	220
11	Naturally regenerating forest without signs of management, incl. primary forests	2563	2414	157
20	Naturally regenerating forest with signs of management, e.g., logging, clear cuts etc.	2128	2076	181
31	Planted forest	207	416	80
32	Plantation forests (rotation time up to 15 years)	82	132	49
40	Oil palm plantations	17	49	25
53	Agroforestry	703	910	153

*CI – confidence interval.

We have calculated and compared the areas of mapped forest management classes with official FAO FRA statistics for the year 2015^37^. Our definitions, provided in Table 1, are in line with the FAO FRA forest definitions. Specifically, naturally regenerating forests (as referred to the FAO FRA), correspond to our classes 11 and 20, planted forests (as called in the FAO FRA) correspond to the mapped classes 31 and 32. The only exception is semi-natural forests, which belong to planted forests in the FAO FRA. Moreover, we used the forest mask with a 10 percent threshold to align our map with the FRA forest definition. However, from the confusion matrix it follows that the mapped forests are overestimated. Therefore, we have also clipped the forest classes by applying 15% and 25% thresholds and then calculated areas, respectively. Table 6 provides forest areas by the FAO FRA and forest areas calculated using 3 thresholds (10%, 15% and 25%). Table 6 includes data at global level and for the 8 countries with the highest forest coverage. Statistics for all other countries could be found in the Data record^38^. Note that one should interpret all the areas with caution since there are discrepancies in the FAO FRA reporting by countries^39^.

**Table 6:** Area comparison of the mapped classes and FAO FRA statistics for 2015.

Country	Naturally regenerated forest, 10^6^ ha				Planted forest, 10^6^ ha			
	FAO FRA	Forest >10%	Forest >15%	Forest >25%	FAO FRA	Forest >10%	Forest >15%	Forest >25%
Russian Federation	795	972	947	910	20	26	25	23
Brazil	494	486	480	465	10	10	10	9
Canada	331	466	441	406	16	4	4	4
United States of America	284	322	307	289	26	33	33	33
Democratic Republic of the Congo	132	184	179	172	0	0	0	0
China	131	155	151	145	79	88	85	80
Australia	131	155	138	121	2	3	3	3
Indonesia	90	128	127	126	5	2	2	2
Global	3792	4680	4361	4044	290	288	279	267

Overall, at global scale, our estimates of forest areas are much higher than those reported in the FAO FRA. This is in line with the study by Bastin *et al*.^40^, who has demonstrated previously that there are 467 million hectares of forest in drylands that were not reported previously (e.g., see Australia in Table 6). We assume that there are also open forests (tree canopy >10% and less than 25%) in Northern latitudes that were not counted previously. For example, other RS-based research^41^ also recognises more forest in Russia (compared to FAO FRA national reporting) due to afforestation of abandoned arable land, expansion of forest to high latitudes and altitudes, accounting for trees in urban and cropland areas. Hence, an additional study should be undertaken to explore this finding further, especially for Russia and Canada. We show much less of the planted forests in Canada, which could be explained by the short time series used in the classification. For the Democratic Republic of the Congo and Indonesia, we show more forested areas due to the confusion between our class 20 and agroforestry. For Brazil, the mapped areas and the FAO FRA reported areas are in line.

## Usage Notes

The reference data set is a unique global data set with forest management information, based on collective expert knowledge. It could be used in various biodiversity and forest related research applications, including mapping forest management at regional and local scale or the analysis of forest drivers and land use assessments.

The global forest management map is provided in spatial geoTiff format (see Data Records section) at ~100 m resolution globally. The map is spatially consistent with tree cover estimates of the Copernicus tree cover product^25^ with 10% threshold applied, thus facilitating comparison and overlays. Users could apply more strict thresholding if needed for their applications. We expect the global forest management map to be especially useful for broad-scale assessments of forest ecosystems, global land use modelling, biodiversity impact assessments, managed forest delineation for carbon accounting and intact forest observation.

Data from the forest management layer have already been used for the mapping of plantations in global terrestrial habitat maps^42^ and in the identification of global areas of biodiversity and climate mitigation importance^43^. However, in general, we recommend using the map for global and supra-regional applications only, rather than at the local scale, owing to the uncertainty in mapped forest management classes when examined locally, similar as to other global earth observation products^44^. For local usage, we would strongly recommend checking the map visually over the area of interest, or to run additional validation. Users may also consider aggregating the map to a coarser resolution.

### Limitations

We would like to remind users about the non-random distribution of the targeted sample, which could be a constraint for some applications. The reference data were collated with the aim of maximizing quality and inter-user agreement rather than an equal spatial distribution.

As a result of independent validation and expert feedback, users should be aware of the following known caveats in relation to the forest management map:

- Planted forests are underestimated, especially, in Canada and Europe.
- Small holder oil palm plantations are mapped as agroforestry.
- Woody and oil palm plantations are underestimated in regions with high cloud coverage and are often confused with naturally regenerated forests (class 20).
- The map extent contains pixels with tree cover fraction of 10%, in line with the CGLS-LC100 collection 2 tree cover fraction layer but not restricted to only forest as land use. Therefore, we would recommend also applying some additional masking if possible.

It should be reiterated that this is a prototype forest management map which can be improved in the future by: (i) using longer time series of RS data and other sources of satellite data, such as Landsat and Sentinel; (ii) improving distribution and increasing in size training data set; (iii) testing other AI methodologies; and (iv) adding more thematic details. Lastly, we stress that is a global product for global applications and should not be used at sub-national level for decision making, at which scale regional maps can provide higher detail and context.

## Supplementary information

Supplementary File 1

Supplementary File 2

## Data Availability

To create the reference data set, including the control and validation data sets, we employed the Geo-Wiki application, which can be used to visually check available land cover and land use maps against very high-resolution imagery. Alternatively, users can employ the LACO-Wiki tool, which has similar functionalities and is openly available, but it requires users to upload the land cover and land use maps. All the geographical operations were done in QGIS 3.8.0, and the accuracy matrixes were calculated in R 3.6.1., using the raster^45^ and dtwSat libraries^46^. The forest management layer was generated using the CGLS-LC100 collection 2 processing line developed by VITO NV on behalf of the European Commission Joint Research Centre (JRC). All code was written in Python 2.7. The regional models are available as Zenodo record^47^, and the related biome cluster are also stored as a separate Zenodo record^48^.
