# Supplementary material for: Global forest management data for 2015 at a 100 m resolution
Source: Sci Data. 2022 May 10;9:199. doi: 10.1038/s41597-022-01332-3 (PMC9091236; doi:10.1038/s41597-022-01332-3)

Gallery of images

Examples and definitions of human impact on forestry landscapes

International Institute for Applied Systems Analysis
Human Impact on Forests Campaign, 2019

Human Impact on Tropical Forests

Content

- Task & Definitions
- Examples
    - Tree age
    - 1 Forest with low human impact
    - 2 Forest with signs of clear cut, logging..
    - 3 Plantations
    - 4 Other landscapes
    - None from the above
- NDVI tool
- Time Series Sentinel Hub

OUR TASK: Identify tree age and level of human impact on forest

Full definitions

1 Forest with very low human impact - forest not affected by human

- **"not disturbed"** - natural forest without any disturbances within the blue box and in the distance of **500 m** (5 blue boxes) in any direction from the blue box
- **"with human impact nearby" -** forest **in the blue box is not disturbed**, but there are roads situated nearby (**within 500 m in any direction from the blue box**), or minor human activities (houses, small agricultural fields) outside of the blue box (within 500 m around).
- **"abandoned crops/pasture" -** blue box area used to be cropland or pasture. Nowadays it is left abandoned for more than 5 years and there are sings of natural afforestation. There are no active cropland/pasture in surroundings.
- **"degraded or disturbed"** - no human activities in the blue box or nearby. Forest is disturbed due to wildfire, windthrow, flooding or insect/diseases outbreaks.

2 Forest with signs of clear-cut, selective logging and forest replanting - managed forest with signs of logging or clear cuts in the blue box or nearby

- **"naturally regrow forest (incl. selective logging)"** - forest is managed (signs of logging in the blue box or in surroundings), but no planting.
- **"replanted forest"** - forest is managed and there are planting activities in the blue box.
- **"regeneration type is not clear"** - forest is managed, but we are not sure if it is planted or naturally regenerated.

3 Plantations - short rotation timber plantations (15 years max) or fruit trees

- **"woody plantations"** - short rotation (15 years max) timber plantations
- **"fruit trees (olives, apples, nuts, cocoa, etc.)"**
- **"oil palm (or other palms)" -** palms have very distinguishable crown shape.
- **"not sure if tree crops or woody plantations" -** in case we cannot distinguish between timber and fruit plantations

4 Other landscapes - trees in agriculture or urban environment

- **"Tree shelter belts, small forest patches" -** group of trees on cropland/pastures in lines or patches
- **"Agro-forestry/sparse trees on agriculture fields" -** individual trees on cropland or pasture, or mixed crops (including trees)
- **"Shifting cultivation"** - a form of agriculture, in which an area is cleared of vegetation and cultivated for a few years and then abandoned for a new area until its fertility has been naturally restored. Usually you can see pieces of land with all the stages of this process.
- **"trees in urban/built-up areas"** - buildings or infrastructure dominant the blue box or surroundings.

These 4 themes represent different levels of human impact on forests:

1 Forest with very low human impact

Intact, primary forest. It is forest where biodiversity is not disturbed by human. There might be some paths or roads in the forest, but wild animals live there (almost) not bothered by people.

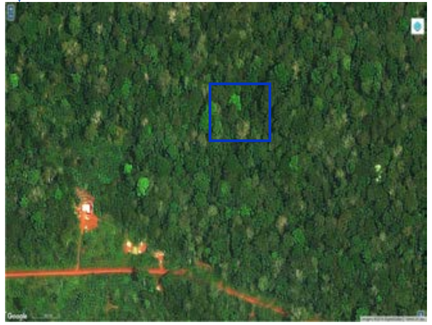

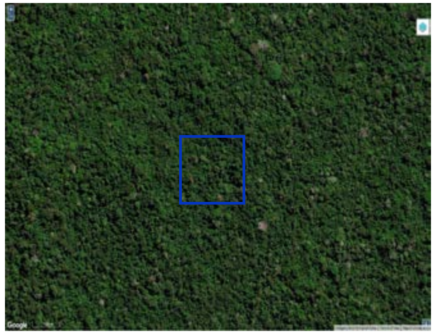

2 Forest with signs of clear-cut, selective logging and forest replanting

Human activities are visible. Parts of the forest have been cut, you can see selective logging. Because of clearcut or logging, the forest then can be naturally regrowing or replanted. Wild animals still live there and find food, but their natural habitat is disturbed.

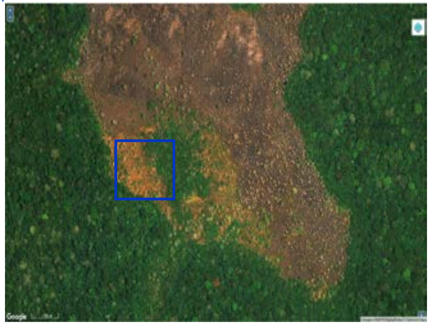

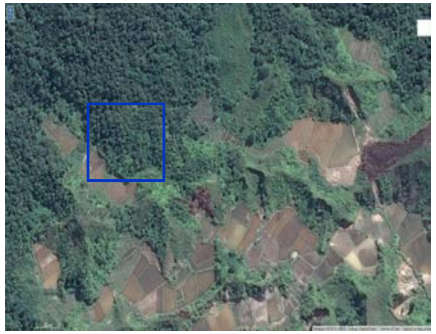

3 Plantations

Plantations are forests with very high human impact. Natural forests have been cleared in order to plant trees under cultivation. The natural environment of animals is replaced.

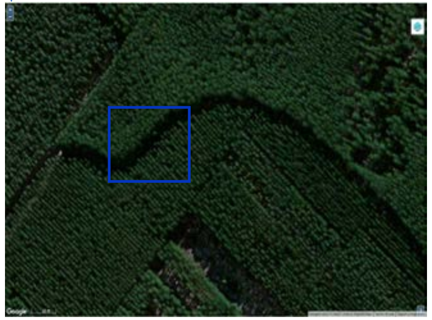

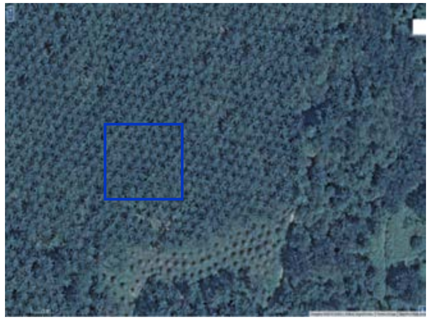

4 Other landscapes

Other landscapes also represent very high level of human impact on forests. They can look like scattered trees on agriculture fields, houses built within the forest or trees in urban areas.

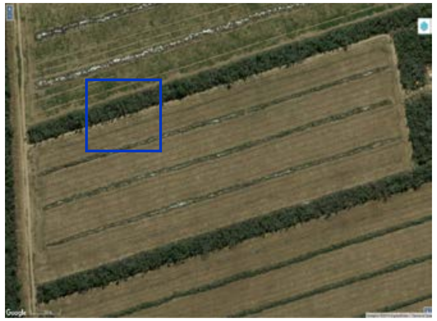

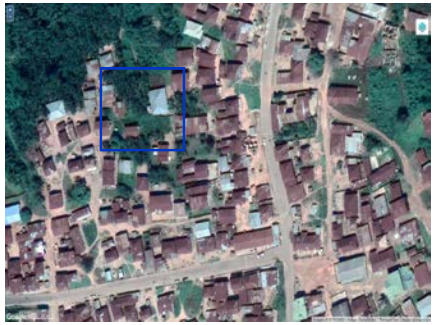

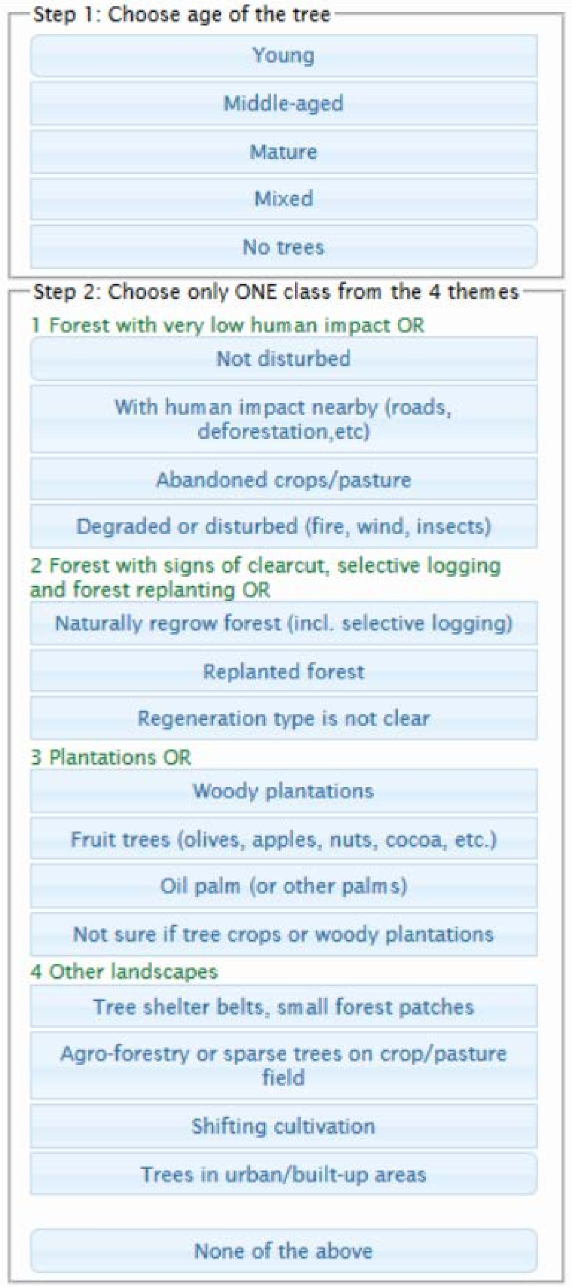

Step 1: Choose age of the tree
- Young
- Middle-aged
- Mature
- Mixed
- No trees

Step 2: Choose only ONE class from the 4 themes

1 Forest with very low human impact OR
- Not disturbed
- With human impact nearby (roads, deforestation,etc)
- Abandoned crops/pasture
- Degraded or disturbed (fire, wind, insects)

2 Forest with signs of clearcut, selective logging and forest replanting OR
- Naturally regrow forest (incl. selective logging)
- Replanted forest
- Regeneration type is not clear

3 Plantations OR
- Woody plantations
- Fruit trees (olives, apples, nuts, cocoa, etc.)
- Oil palm (or other palms)
- Not sure if tree crops or woody plantations

4 Other landscapes
- Tree shelter belts, small forest patches
- Agro-forestry or sparse trees on crop/pasture field
- Shifting cultivation
- Trees in urban/built-up areas

- None of the above

Tree age

Visual estimate of the size of trees in comparison with surroundings

Young

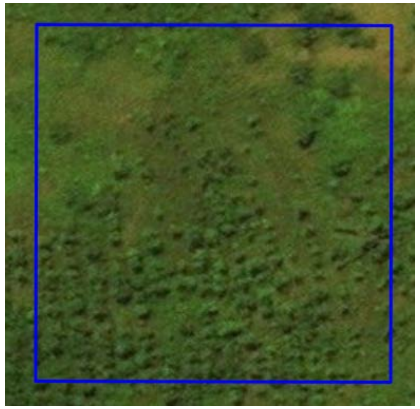

Middle-aged

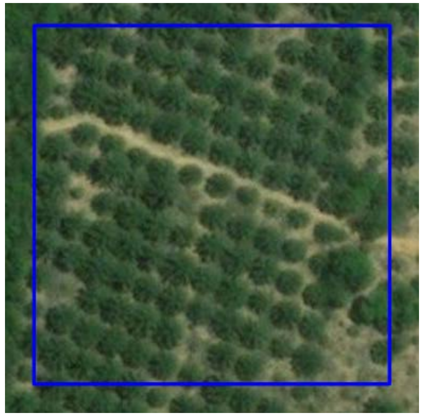

Mature

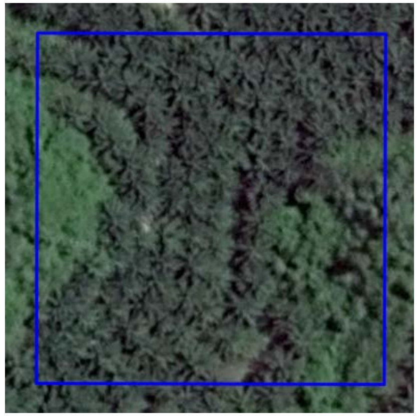

Mixed

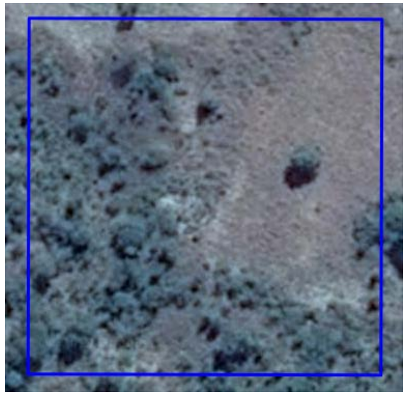

No trees

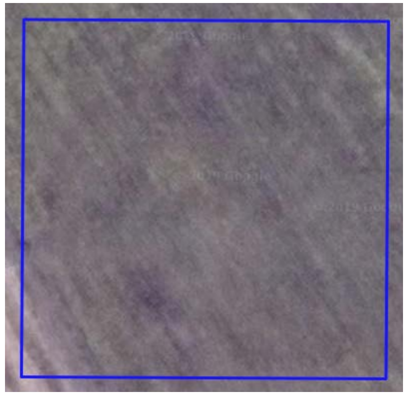

Young

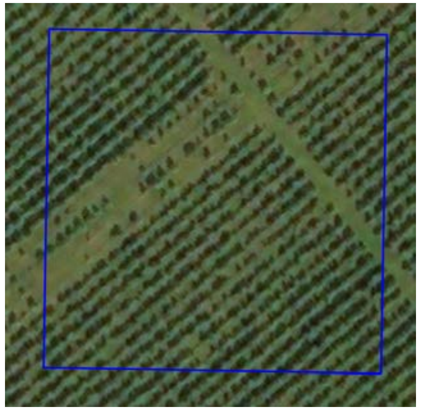

Middle-aged

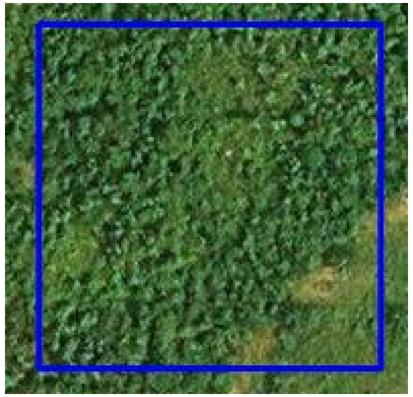

Mature

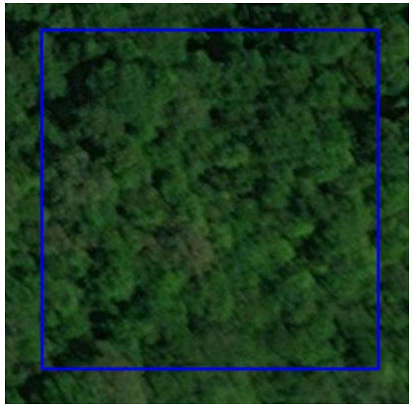

Mixed

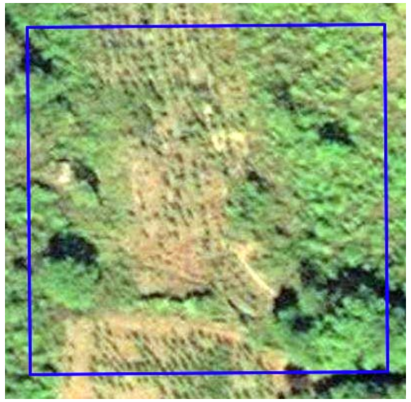

No trees

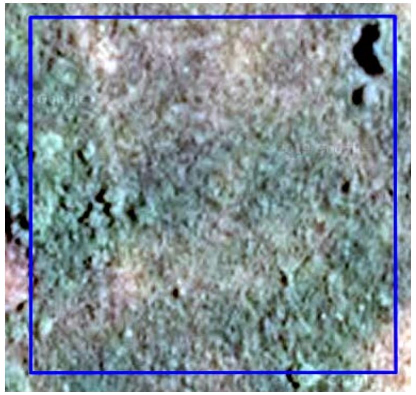

1. Forest with very low human impact

Forest with very low human impact

- Not disturbed
- With human impact nearby (roads, deforestation,etc)
- Abandoned crops/pasture
- Degraded or disturbed (fire, wind, insects)

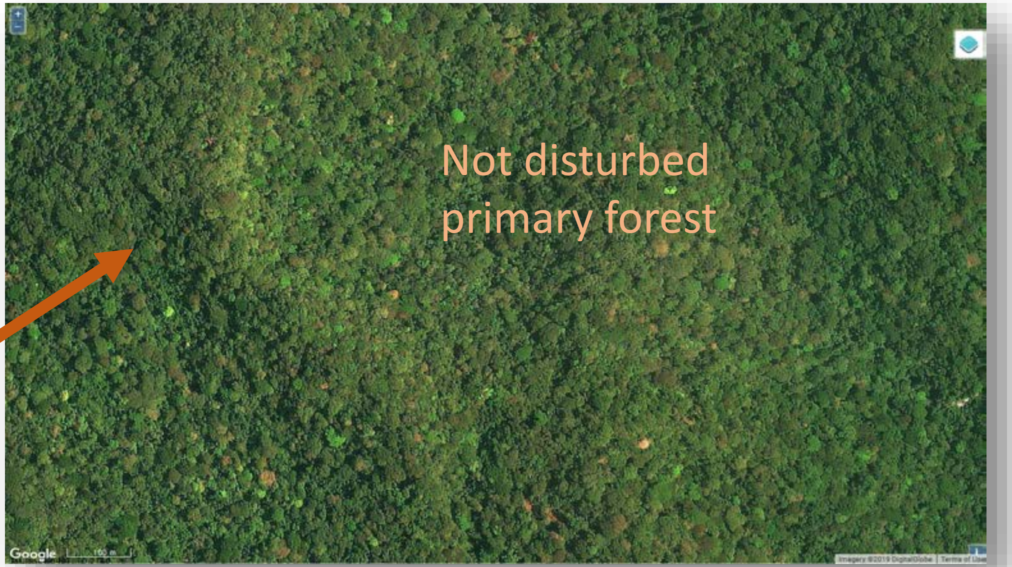

We observe signs of deforestation, but still far away (more than 500 m.) from the blue box. In it the forest is primary, intact forest.

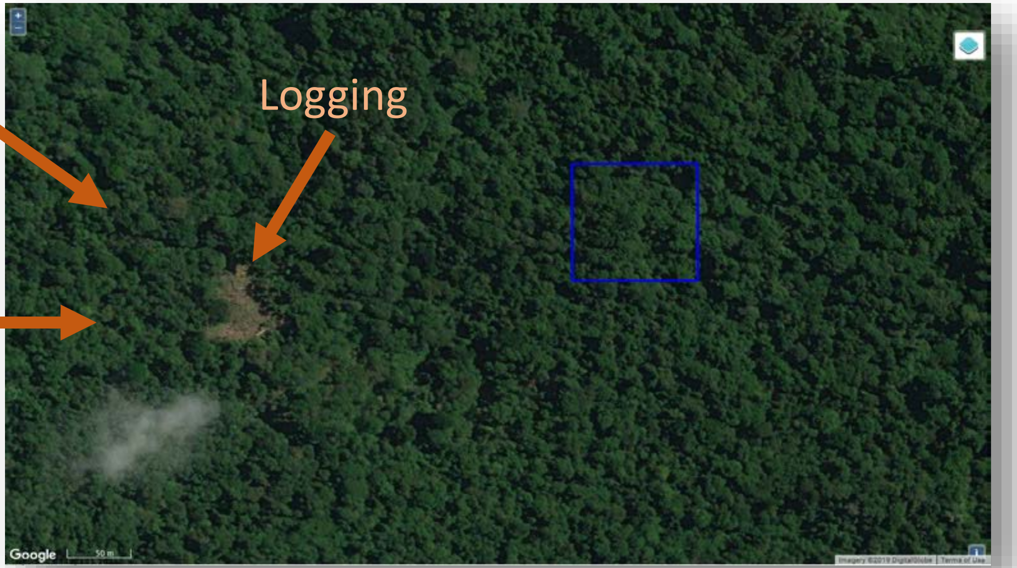

1. Forest with very low human impact

Not disturbed

On this example you can see <u>mature</u> forest in Angola, Africa, which is <u>not disturbed</u>. There are no signs of human interference or non-human disturbance neither in the blue box, nor in the surroundings (in 500 m. distance to all directions). We use the most recent available image from 2017 to prove our decision.

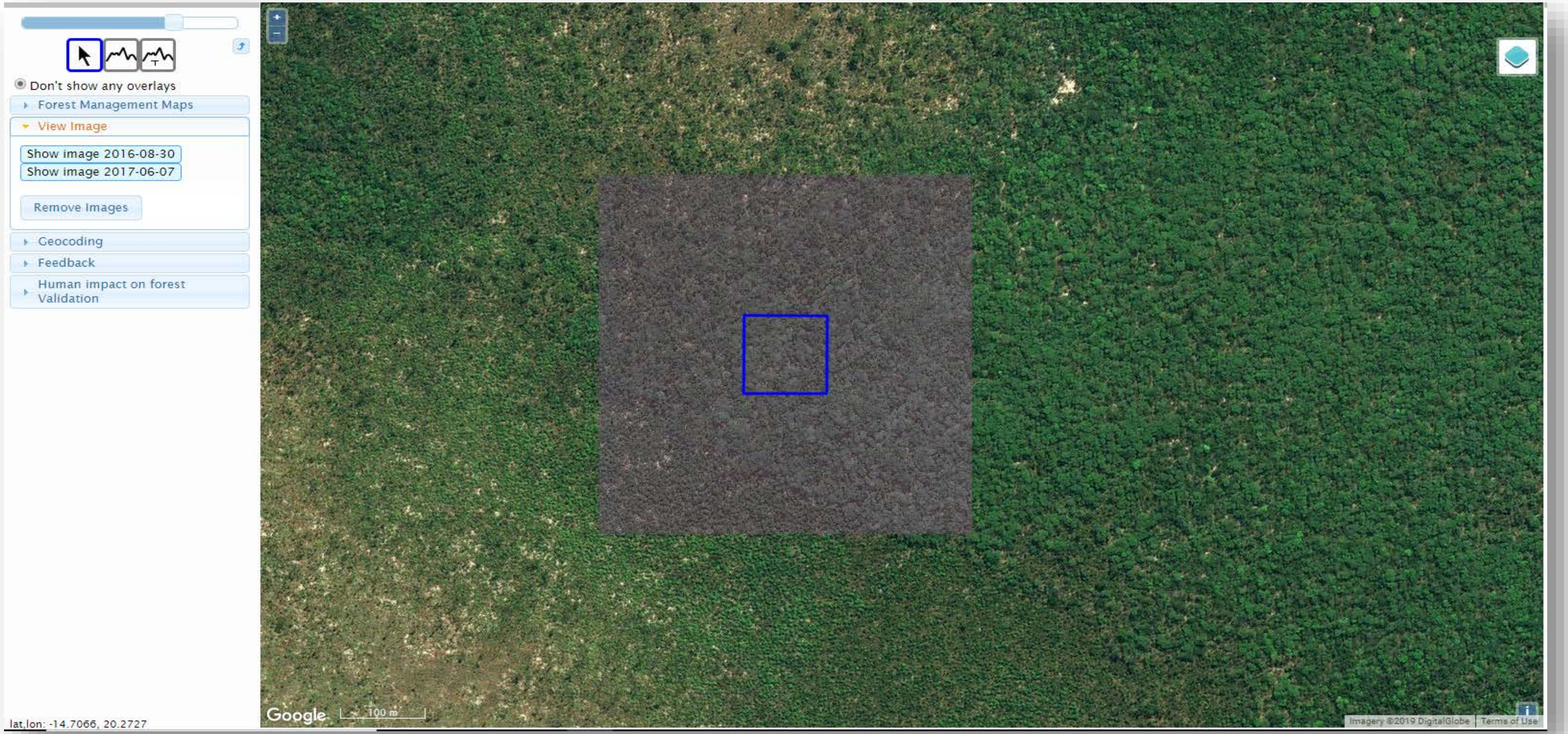

1. Forest with very low human impact
With human impact nearby (roads, deforestation)

On the example below you see mature forest with human impact nearby in Peru, South America. The forest inside the blue box is intact and, on the side, (closer as 500 m. away from the blue box) we see logging.

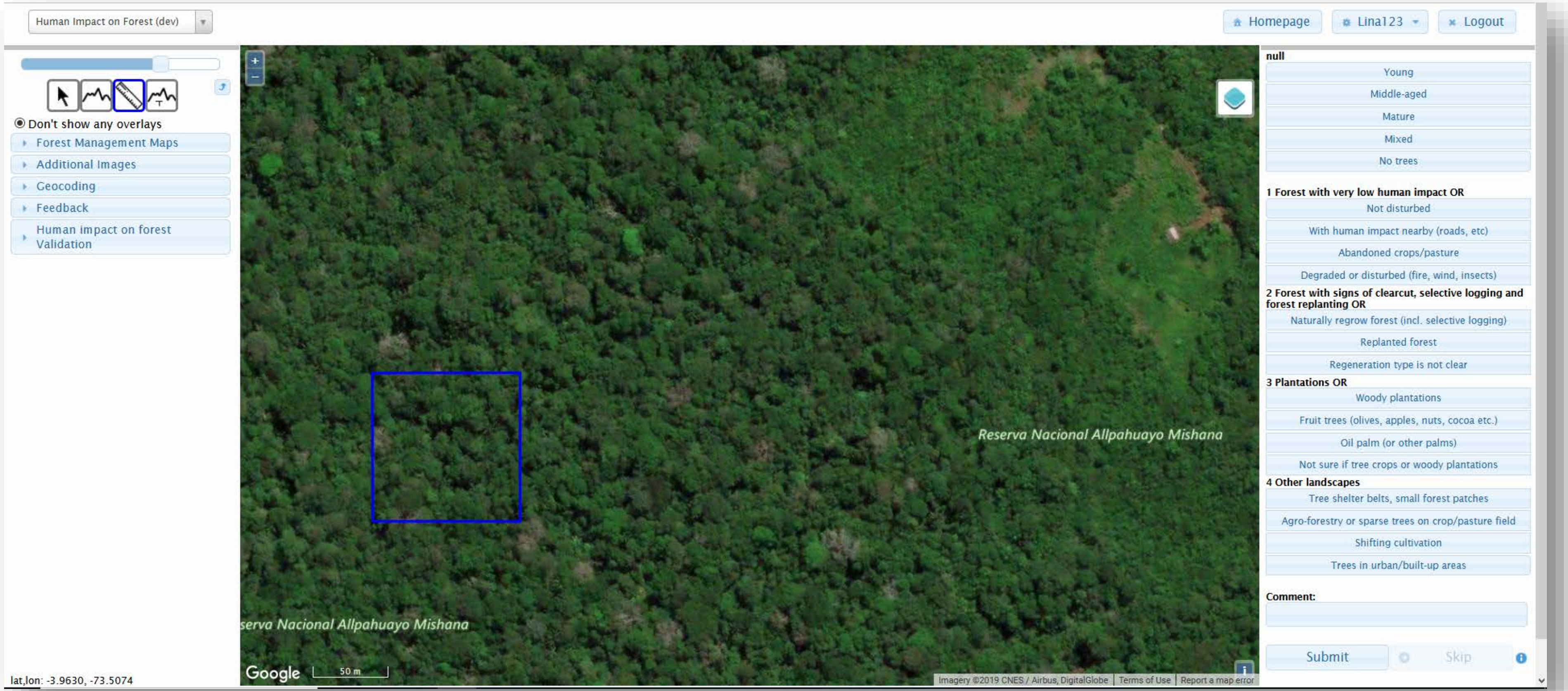

1. Forest with very low human impact

With human impact nearby (roads, deforestation)

You see mature forest with human impact nearby in Argentina. The forest inside the blue box is intact, primary forest. We see road and signs of logging outside the blue box, but closer than 500m. in any direction from the blue box.

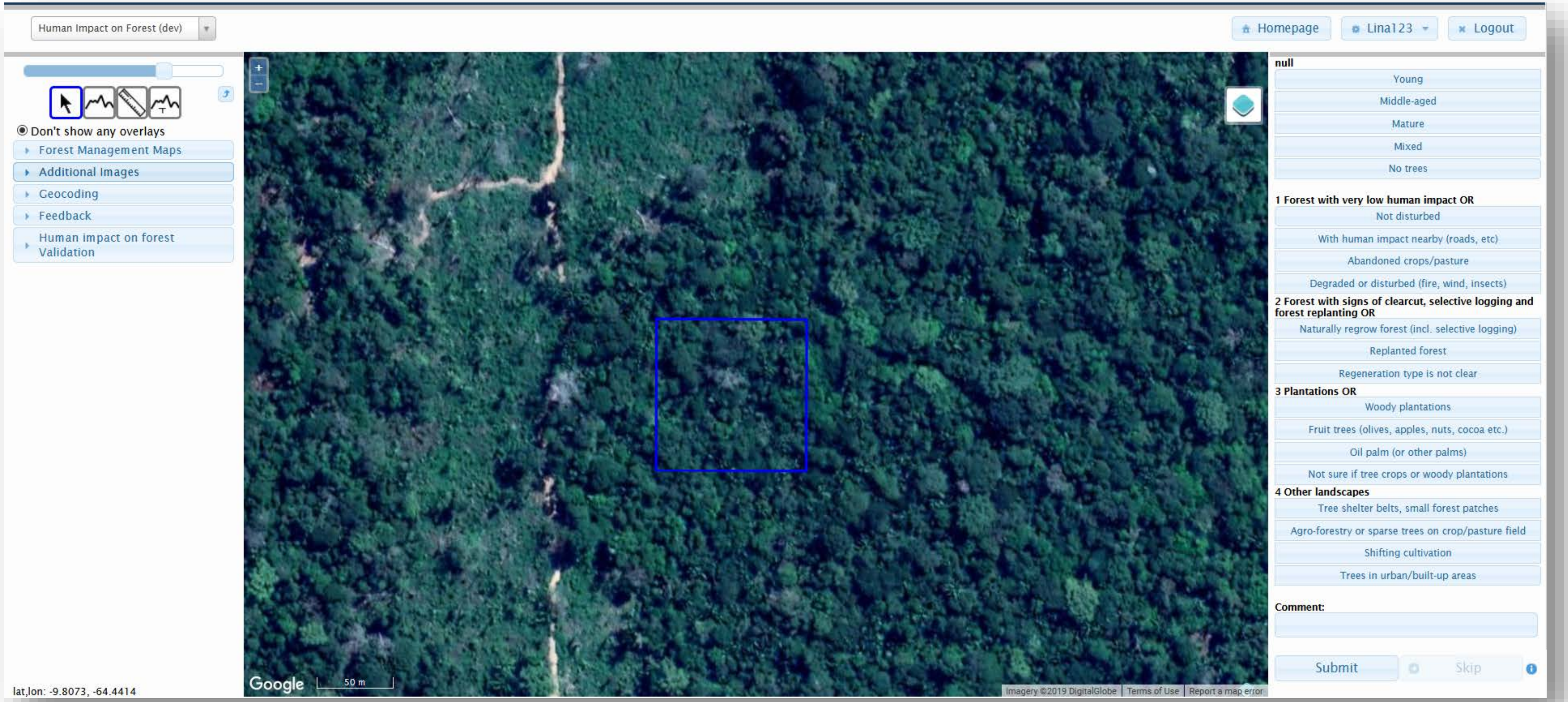

2. Forest with signs of clear-cut, selective logging and forest replanting

Naturally regrow forest

Forest with signs of clearcut, selective logging and forest replanting

- Naturally regrow forest (incl. selective logging)
- Replanted forest
- Regeneration type is not clear

The class “Replanted forest” is rare for tropical landscapes.
The class “Regeneration type is not clear” means that it is visually unclear if forest is naturally regrowth or replanted, so both categories are not very likely for the tropics.

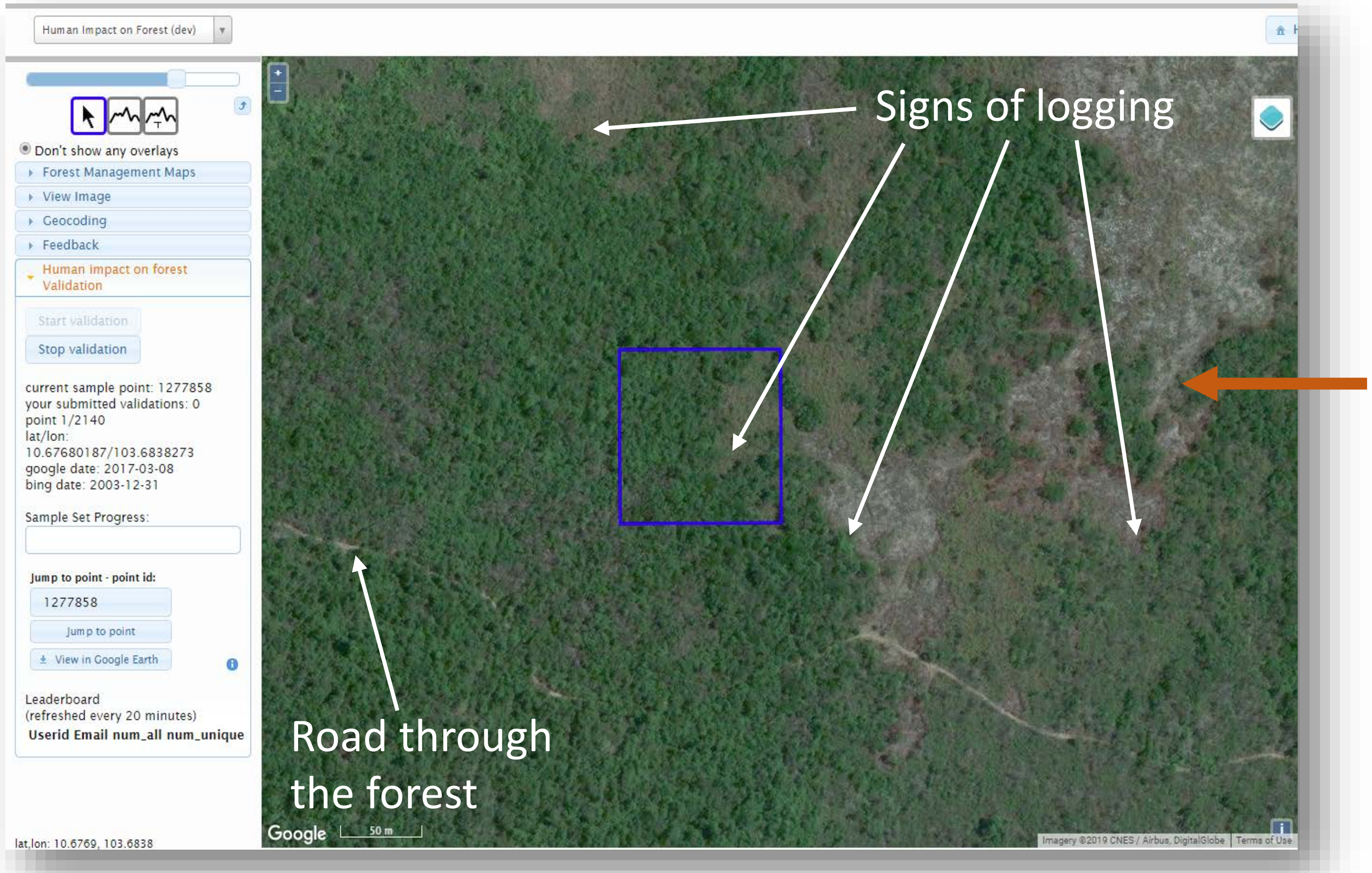

This is an example of naturally regrow forest. This is middle-aged, naturally regrow forest in Cambodia. There are signs of logging, forest roads and forest natural regeneration.

Forest with human impact nearby (roads, deforestation etc.)
is very different from
Naturally regrow forest (incl. selective logging)

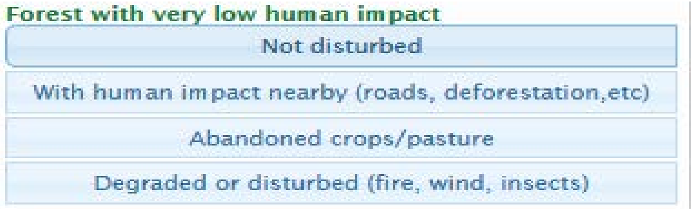

≠

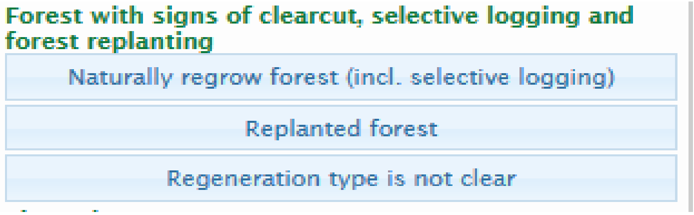

Here you see mature forest in Bolivia. This is forest with human impact nearby, we see clear-cut within 500 m. in some directions from the blue box, but the forest inside the box is intact and primary. Choose this class when you observe signs of human activities not too close to the blue box and no human activities in the blue box. The forest must be primary, not cut, not replanted.

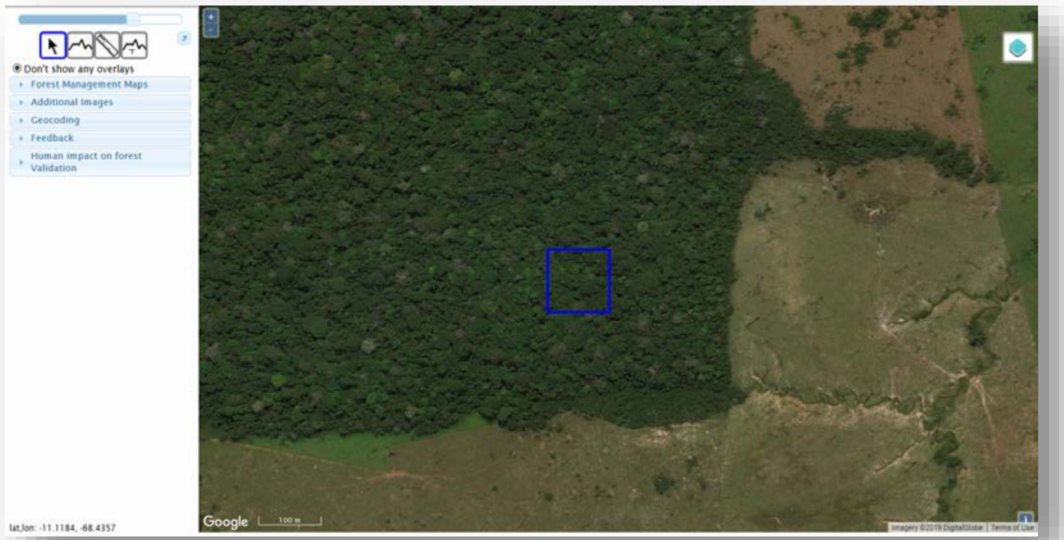

On this image in Brazil, forest was cut. Inside the blue box, new trees are growing but there are a few mature trees, so we identify their age as mixed. This is naturally regrow forest. Very often there are cropland or urban areas close to forests of these types.

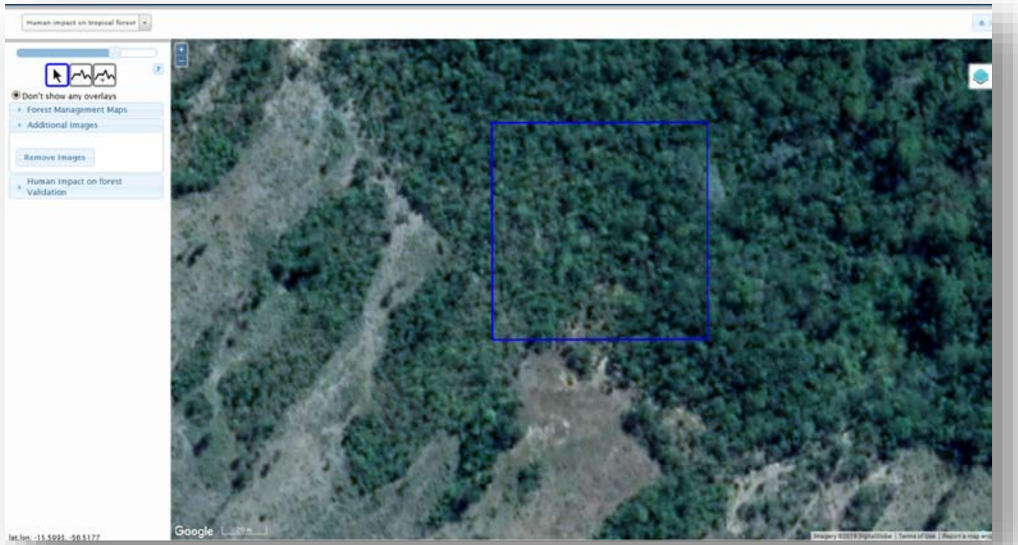

3. Plantations

Fruit trees

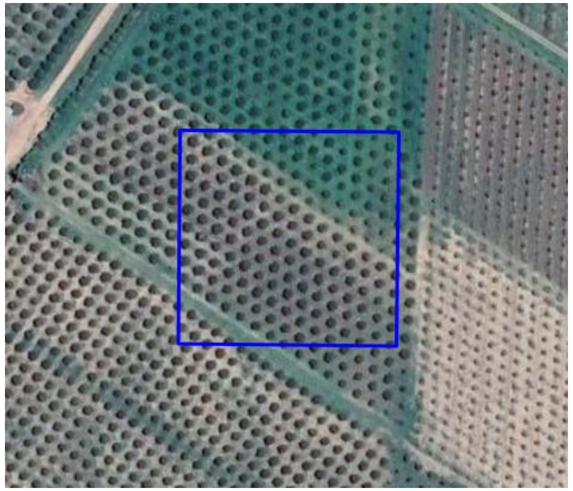

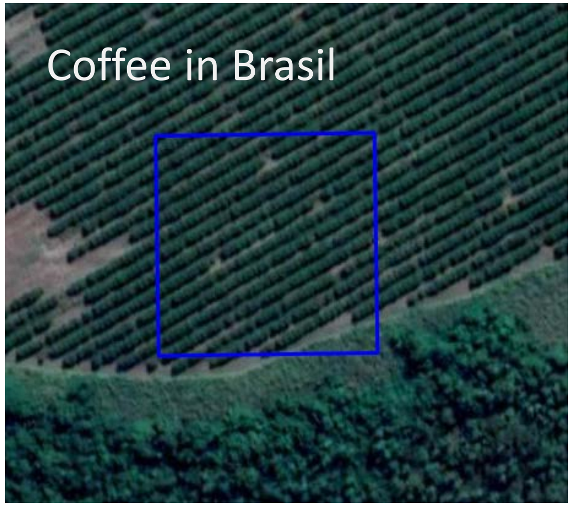

Woody plantation

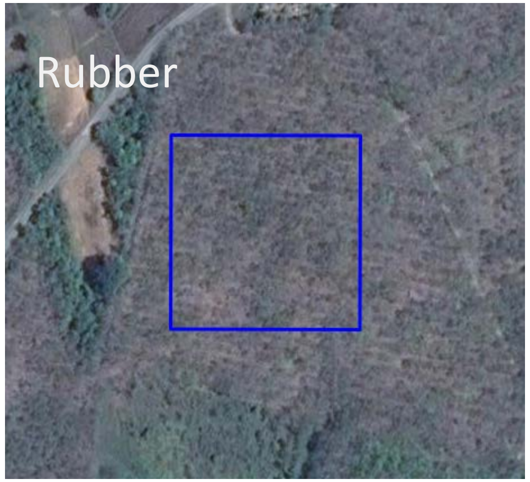

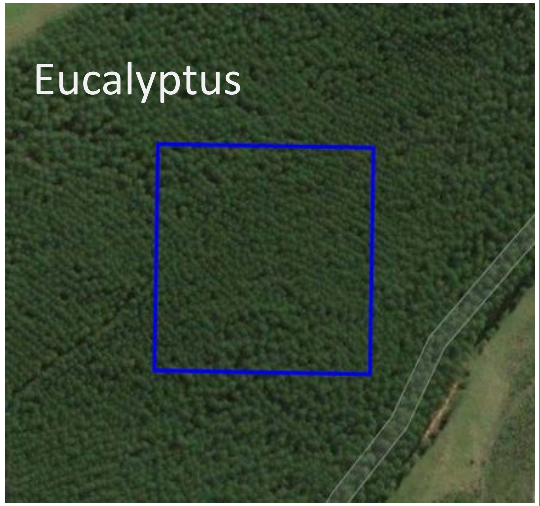

Oil palms

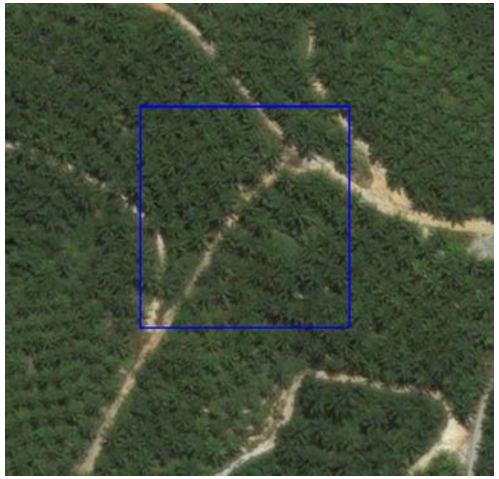

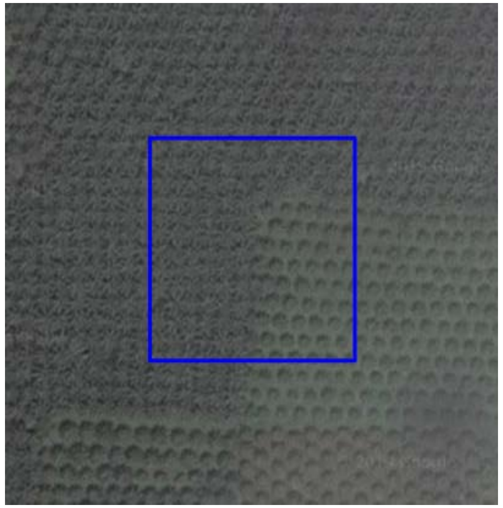

Not sure if tree crops or woody plantation

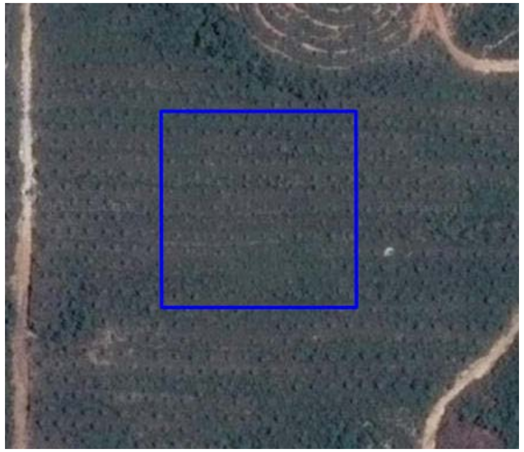

Woody Plantations (eucalyptus)

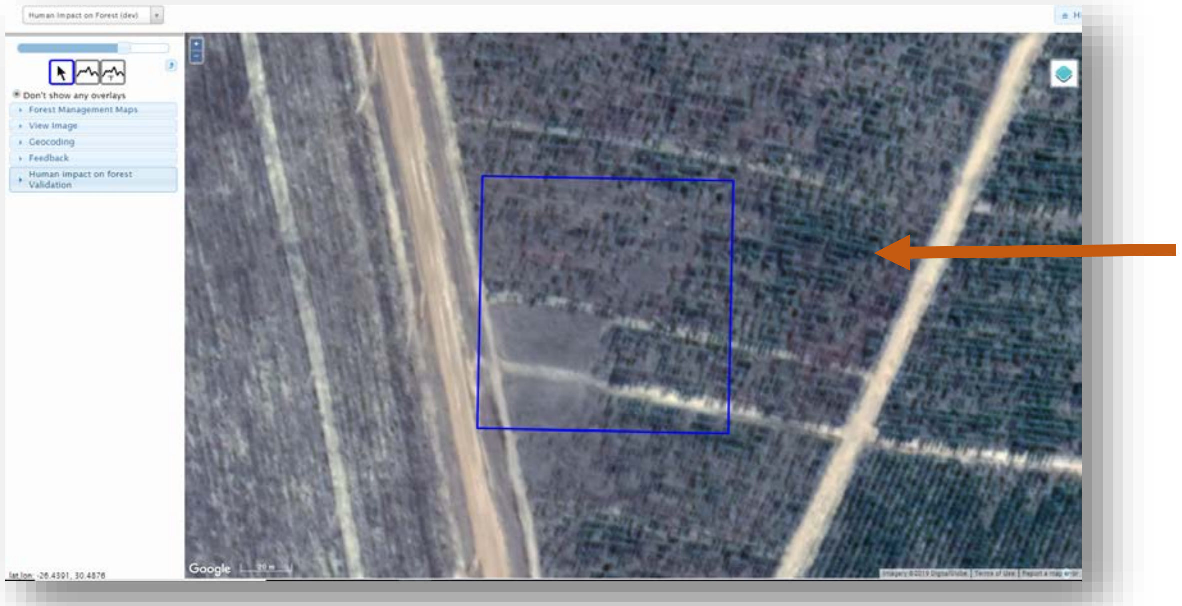

This is an example for a woody plantation from Swaziland, Africa - young eucalyptus trees (very typical for that region).

Below are 4 images from Google Earth for the same location to show the short rotation time, in which eucalyptus trees are cut, replanted and cut again.

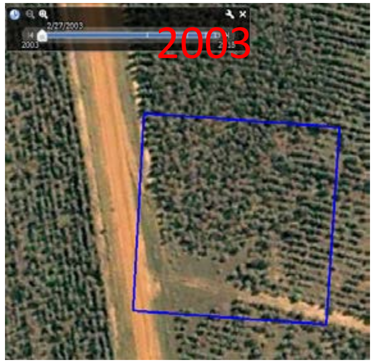

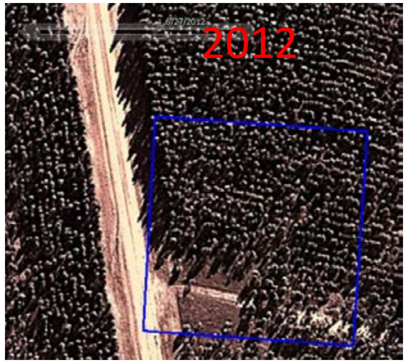

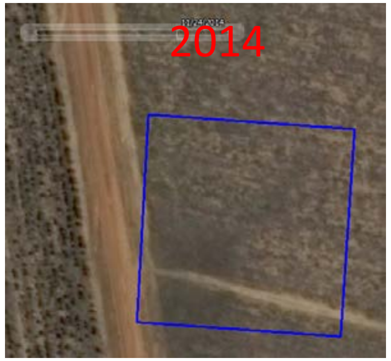

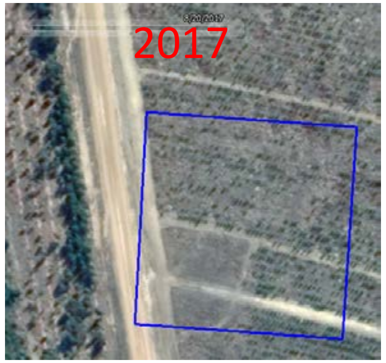

Google Earth Time Series
2003 – young
2012 – mature
2014 – cleaned
2017 – young,
next generation

Woody plantation (mixed)

Example for mosaic landscape with oil palms. The blue square consists mainly of rubber trees, that is why we classify the image as mature, woody plantation, but not as oil palm plantation.

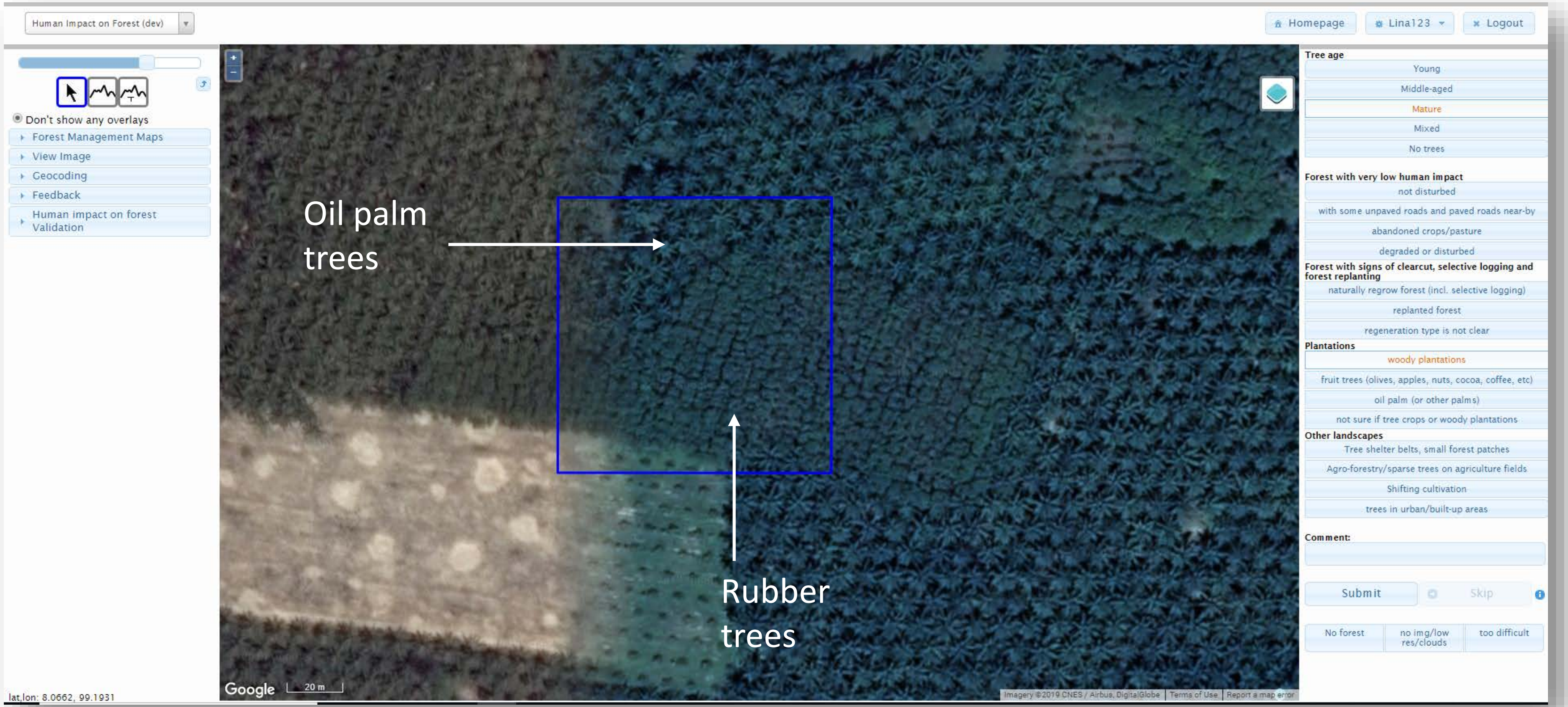

Fruit Tree Plantation

Example for mature, fruit tree plantation. Citrus fruit trees in Guatemala. The trees are not very dense planted, that is why we know that they are fruit trees, but not woody plantations. Shelter belt along the road covers a small portion of the blue box and therefore not accounted.

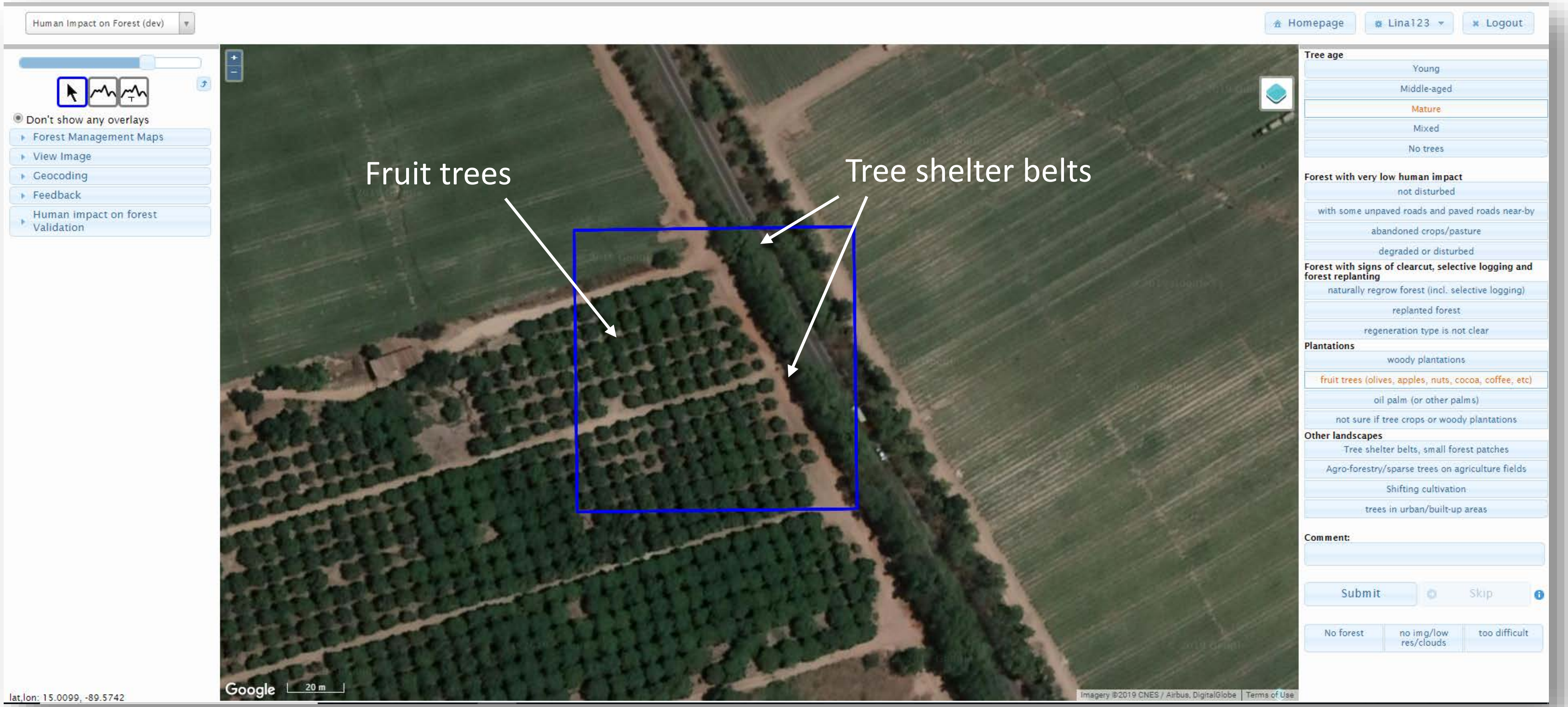

Oil Palm Plantation

This is an example for mature, oil palm plantation in Thailand.

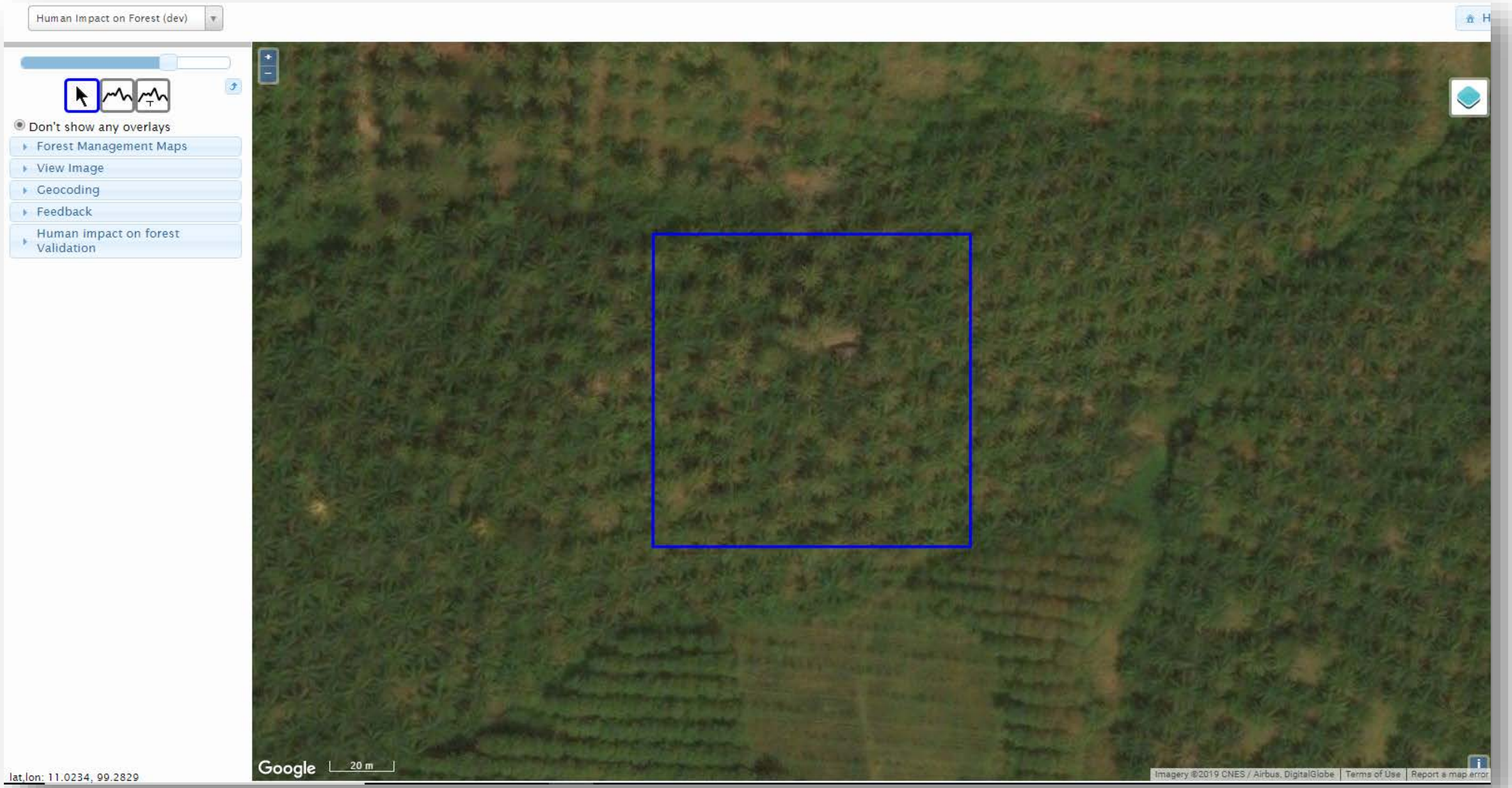

Oil Palm Plantation

Below you see typical landscape for Indonesia. This is oil palm plantation, with mature tree age.

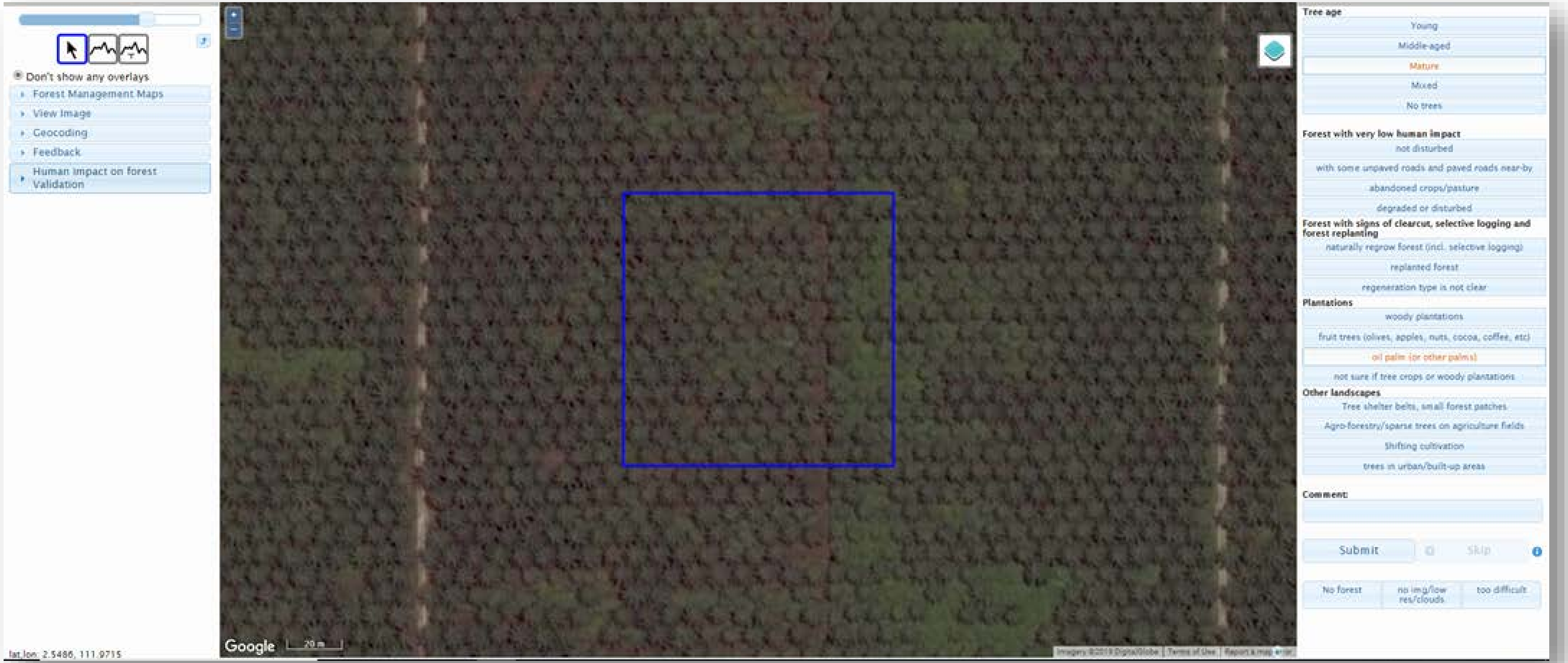

4. Other landscapes

Tree shelter belts, small forest patches

Below you see tree shelter belts with mature tree age in Bolivia.
Tree shelter belts are corridors of trees less than 20 meters wide. However tree shelter belts in South America make exception, here corridors can be up to 50 meters wide, or more.

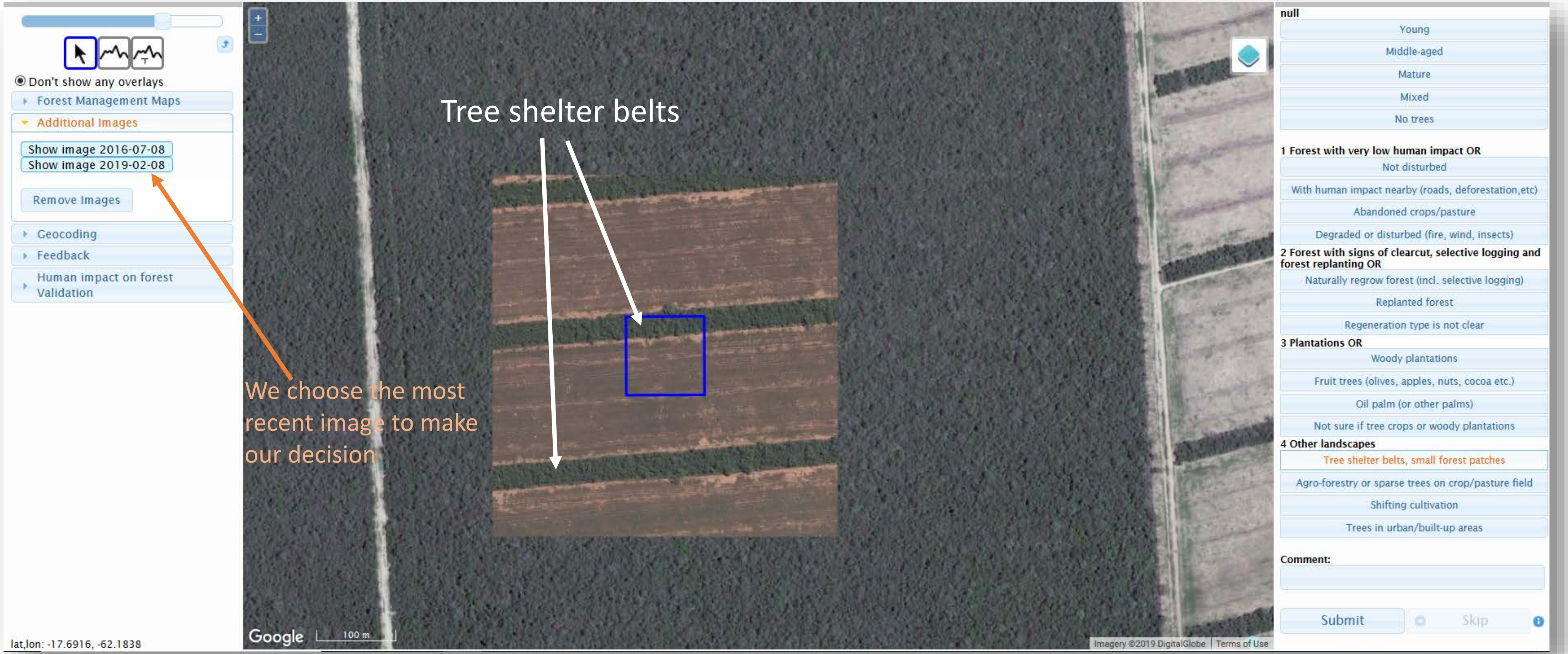

Tree shelter belts, small forest patches

This is an example for small forest patches with mixed tree age in Zimbabwe.

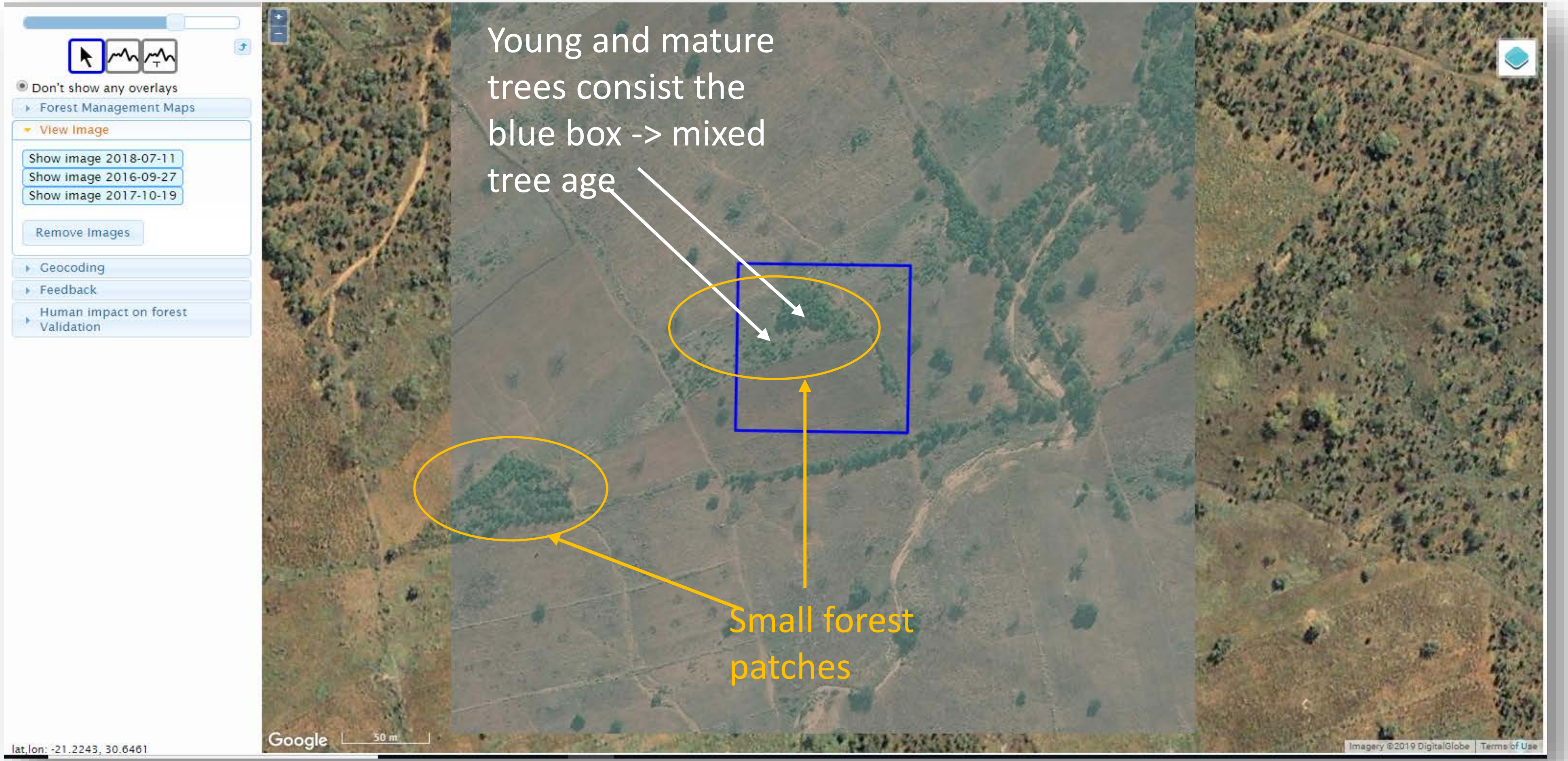

Agro-forestry/sparse trees on agriculture fields

This is an example of <u>agro-forestry</u> in Brazil, <u>mature</u> tree age.

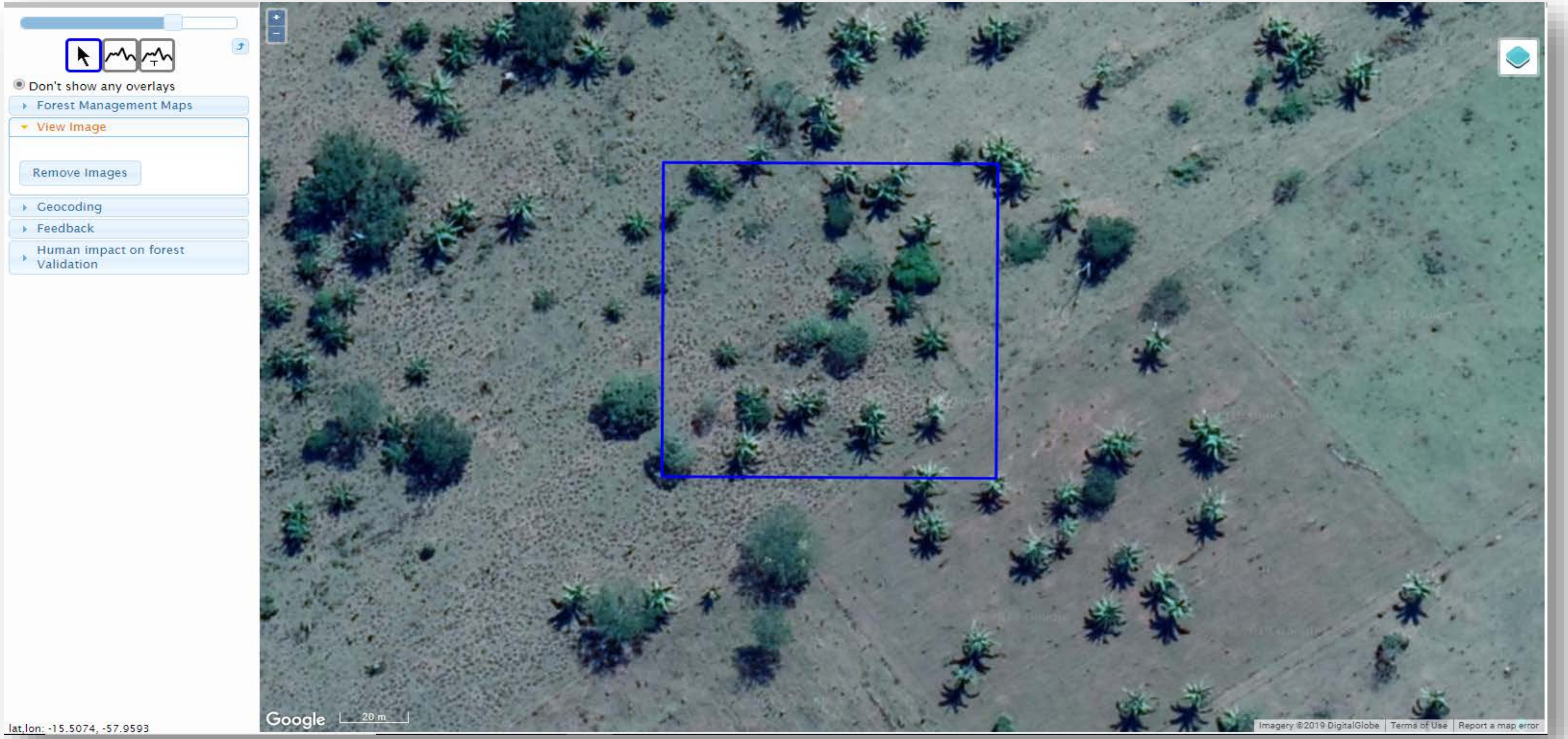

Agro-forestry/sparse trees on agriculture fields

Sparse trees on agricultural fields in Congo, Africa.

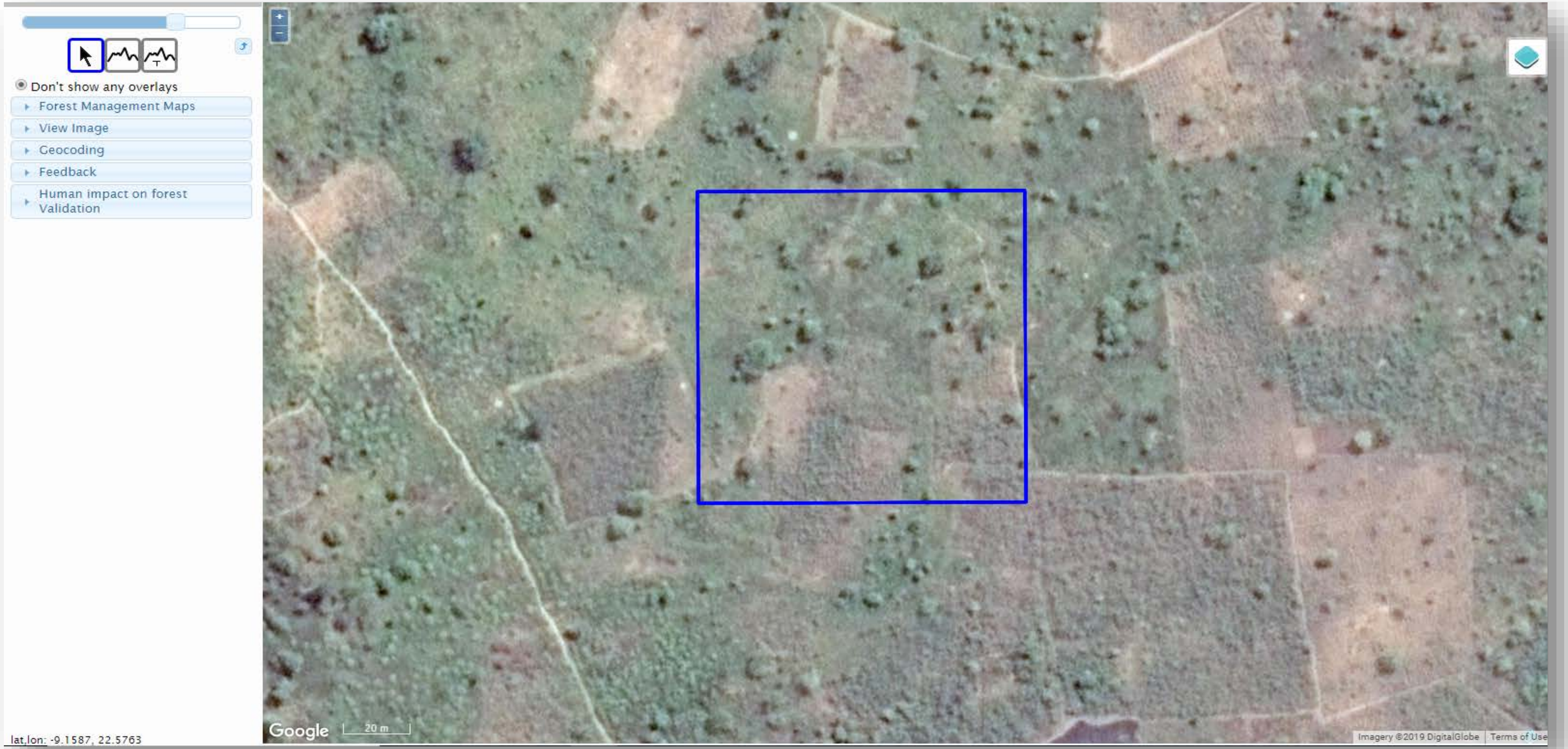

Agro-forestry/sparse trees on agriculture fields

Sparse trees on agriculture fields (pastures), mature tree age, Brazil.

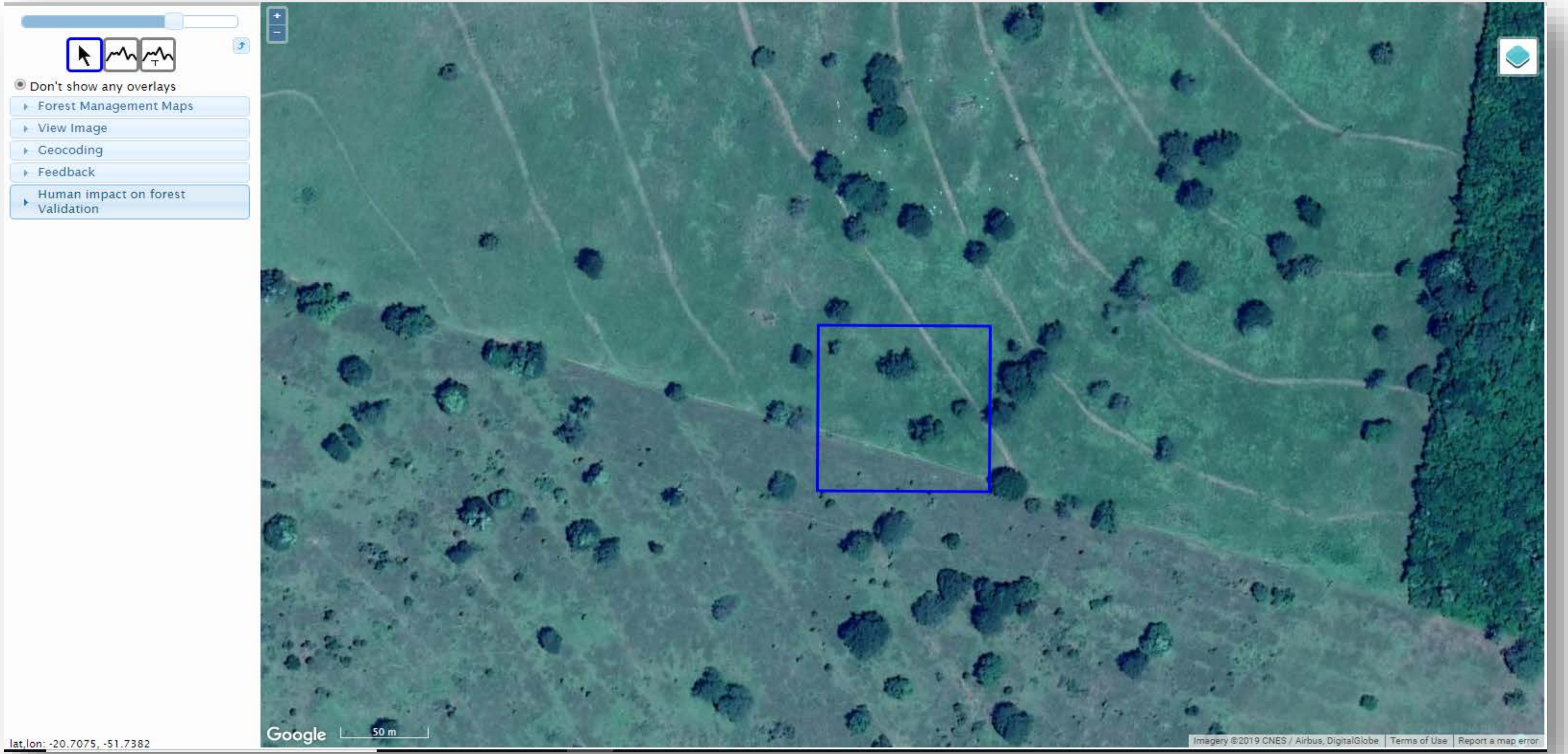

Shifting cultivation

Shifting cultivation in Congo, Africa. Local people recut forest, use the clean area for agricultural activities for a while and then abandon it. We can trace all the changes of land use in the surroundings.

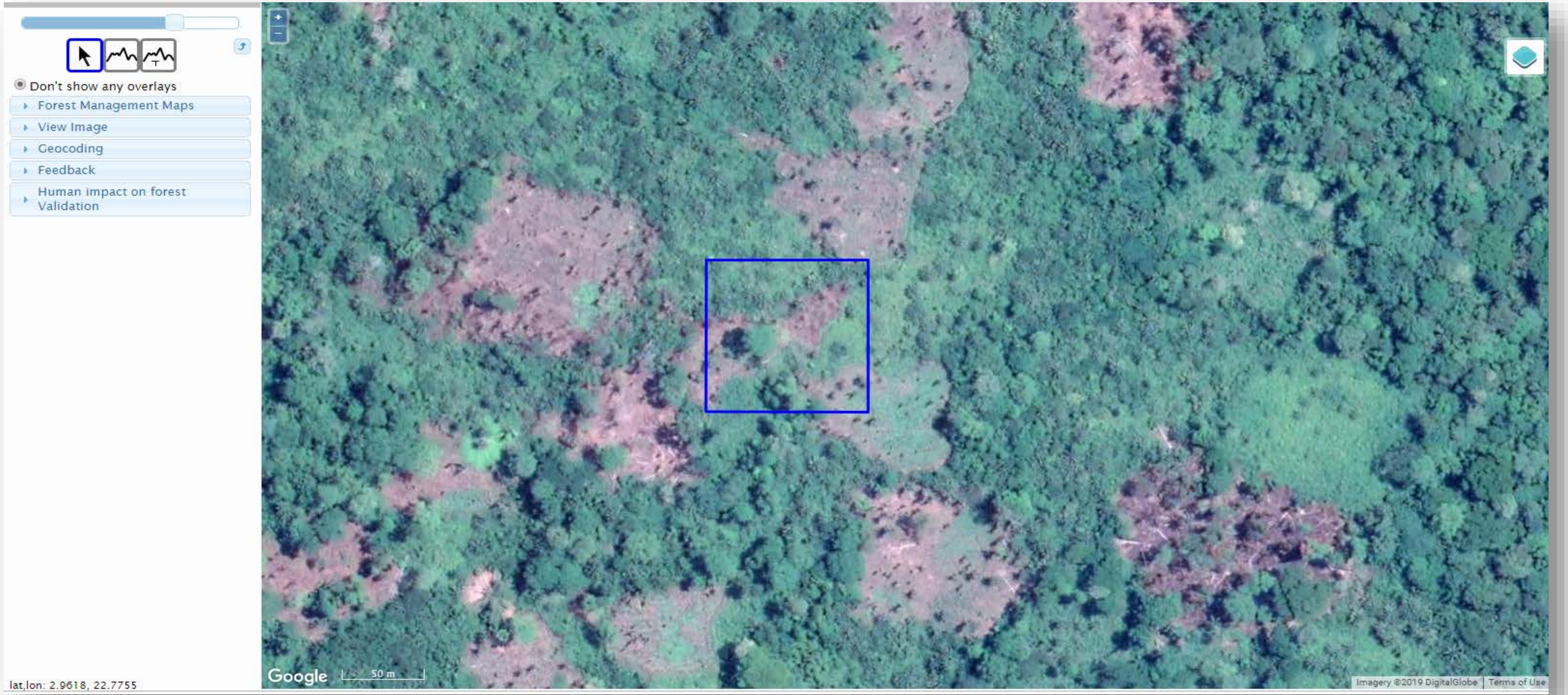

Trees in urban/built-up areas

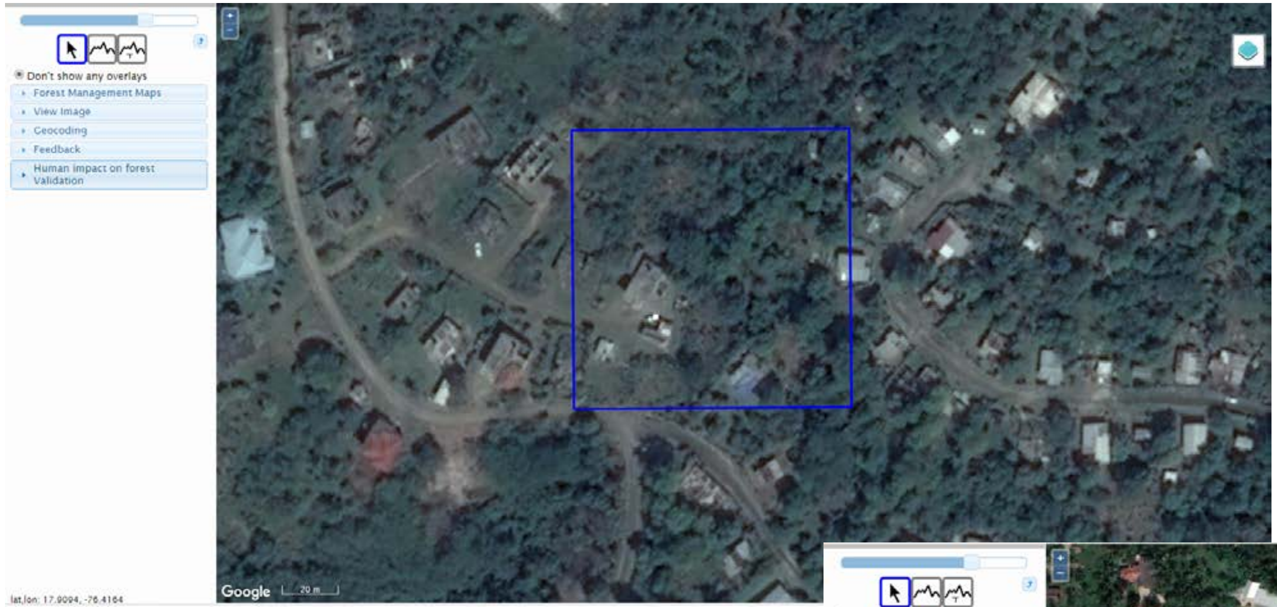

Example for <u>mature</u> trees in <u>urban/built-up</u> areas in Jamaica

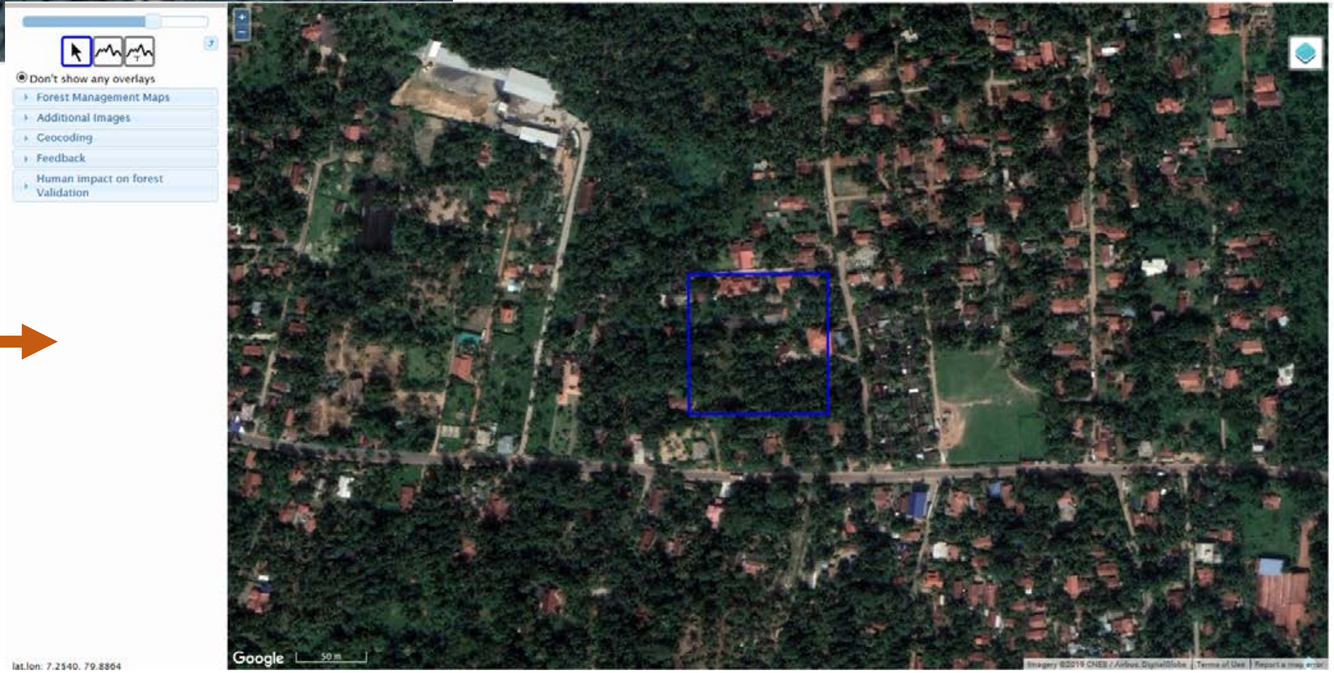

Example for <u>mature</u> trees in <u>urban/built-up</u> areas in Sri Lanka

None from the above

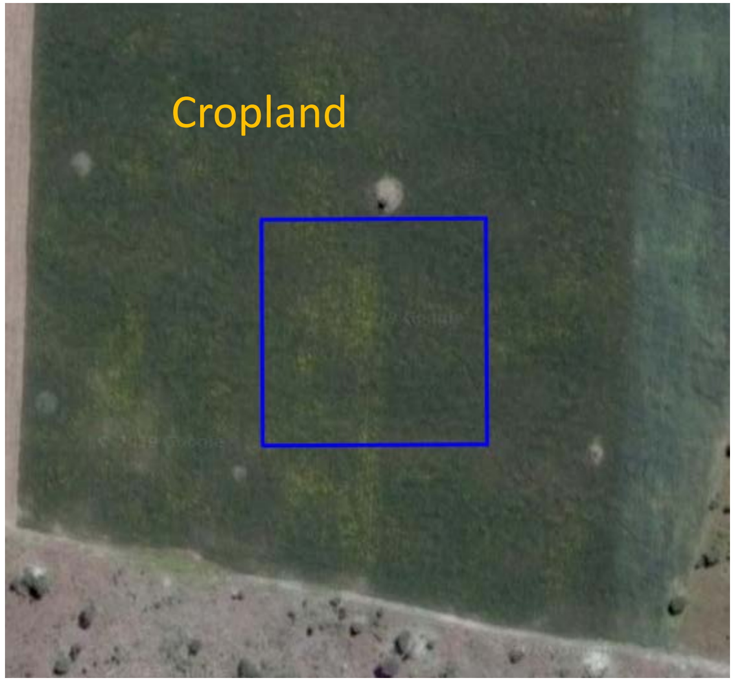

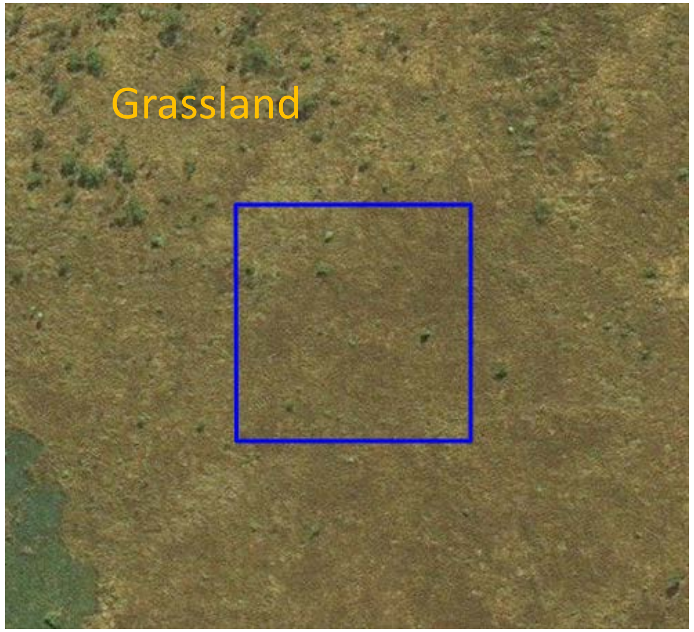

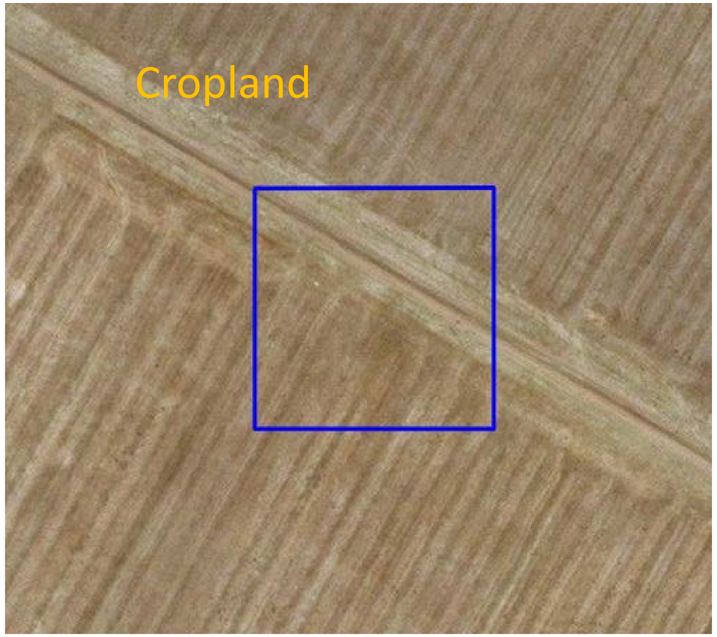

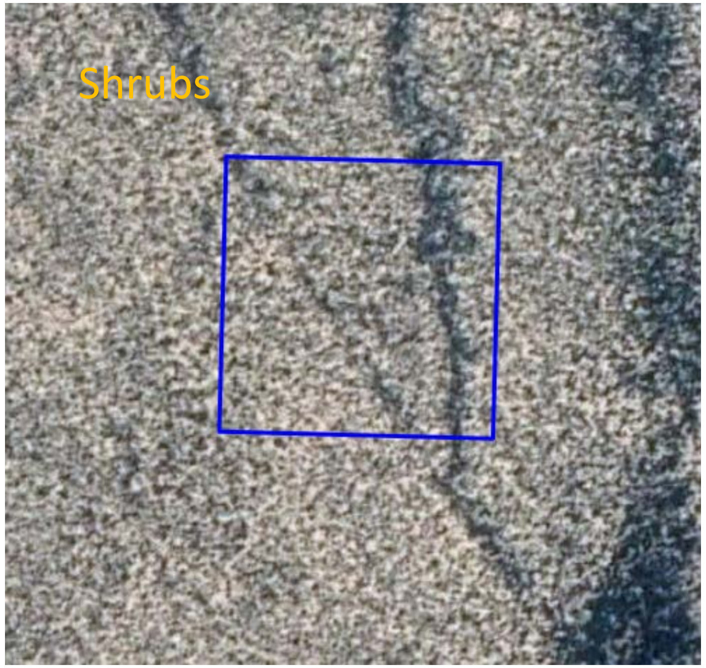

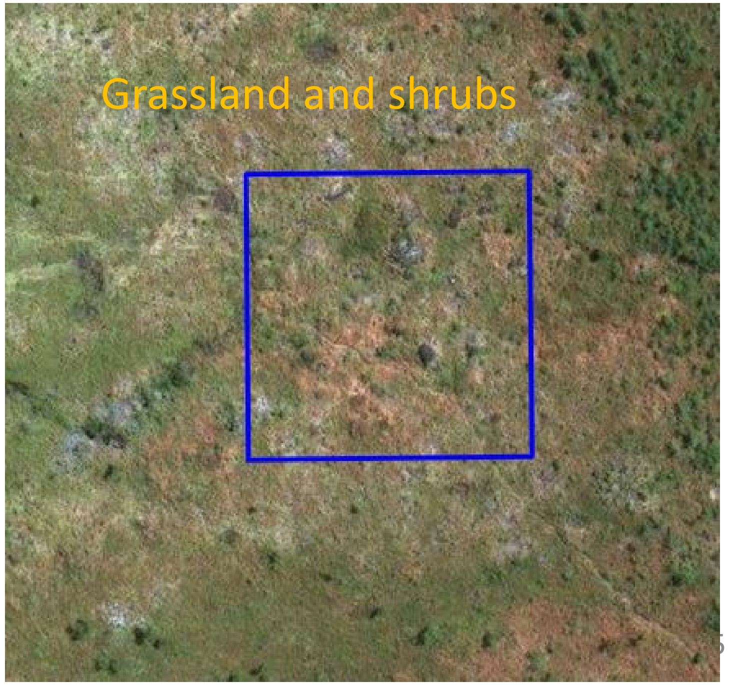

NDVI tool (Normalized Difference Vegetation Index)

In certain cases you can also use the NDVI tool, an index of greenness showing dynamics of vegetation for a certain location. Below is an example of eucalyptus plantations in Africa. Here NDVI indicates the short rotation time of the plantation, less than 10 years (see e.g. 1, 2). The lower values show that the trees were cut in 2002, then eucalyptus trees were replanted, grew mature and in 2009 were cut again and replanted. In 2016 they were already mature - cut again, and replanted - rotation. Historical imageries from Google Earth (see e.g. 3) confirm the same observation.

1

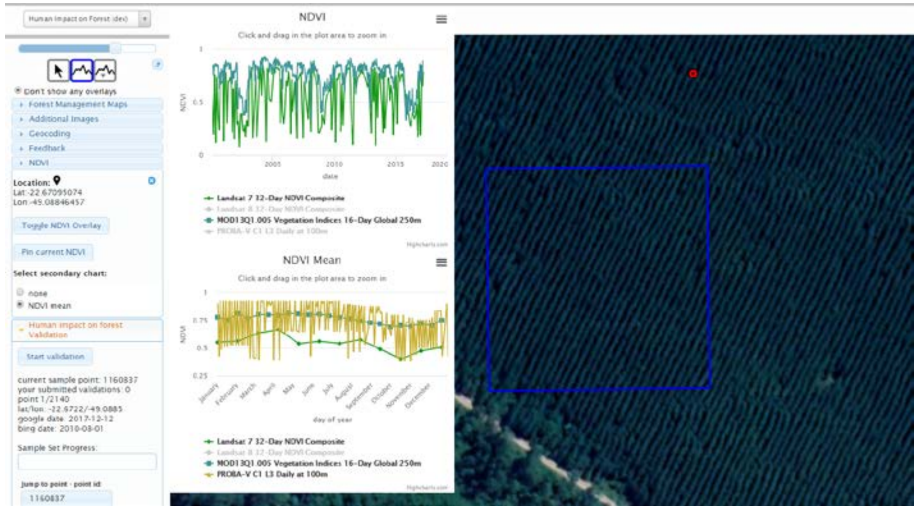

2

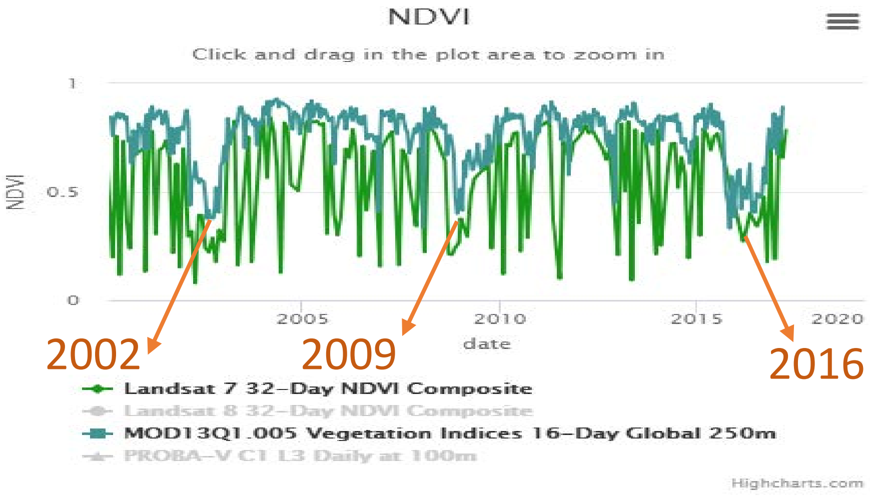

3

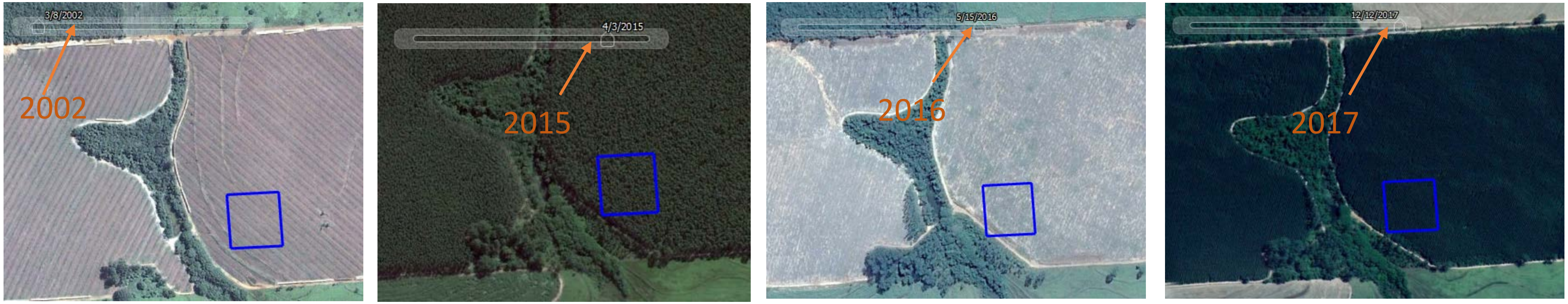

Time Series Sentinel Hub

Another tool which may be useful in certain cases as well, is Sentinel Time Series derived form Sentinel Hub. Below you can see images from 2016 and 2017 during the different seasons in Zambia, Africa. The intensive red color shows that there are mostly green trees or vegetation. There are no major changes in the landscape during 2016 as you can see. In 2017 we observe that forest was cut and big irrigation system was built. We can identify the gradual development of the irrigation system, where people probably pump out water from the river in order to irrigate crops.
In general we use Sentinel Hub to track changes in landscapes.

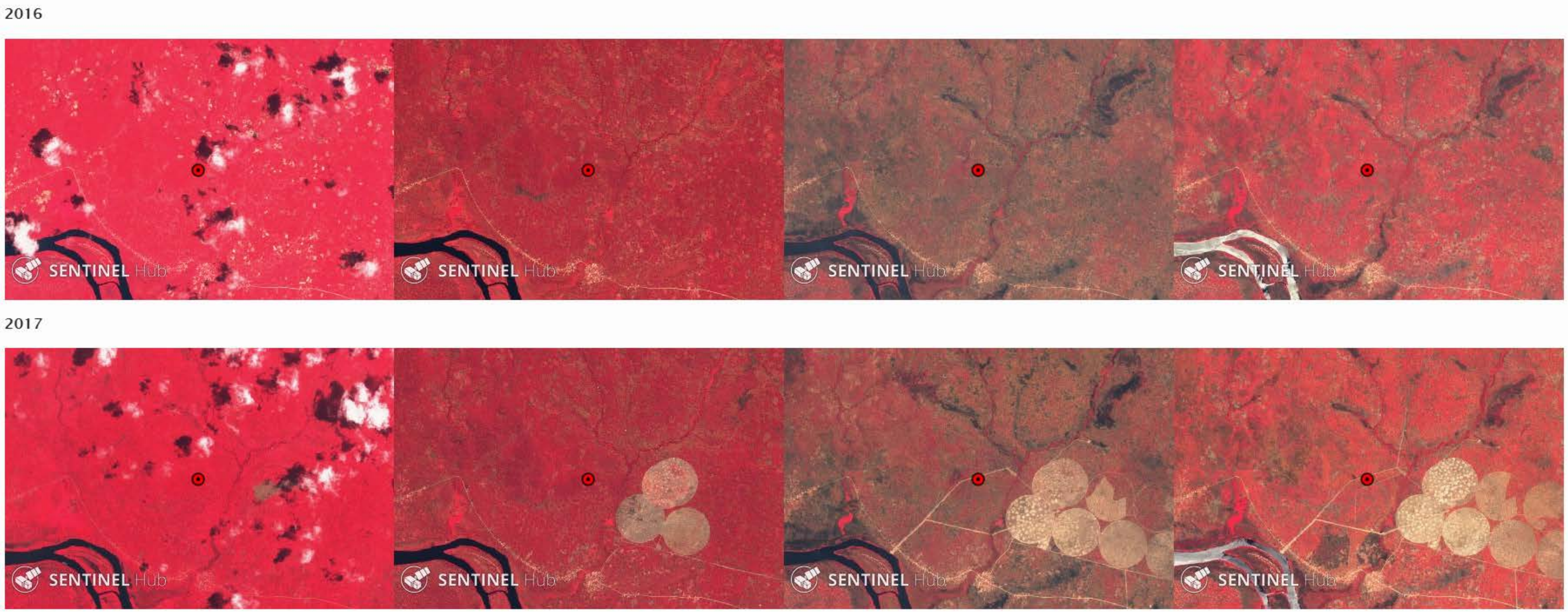

Supplement: Supplementary file 1 [file 41597_2022_1332_MOESM1_ESM.pdf]
