# Supplementary material for: Global forest management data for 2015 at a 100 m resolution
Source: Sci Data. 2022 May 10;9:199. doi: 10.1038/s41597-022-01332-3 (PMC9091236; doi:10.1038/s41597-022-01332-3)
Supplement: Supplementary file 2 [file 41597_2022_1332_MOESM2_ESM.pdf]

Survey among the crowdsourcing campaigns participants (64 responses)

Gender:

- Male: 74 %
- Female: 26 %

Age range:

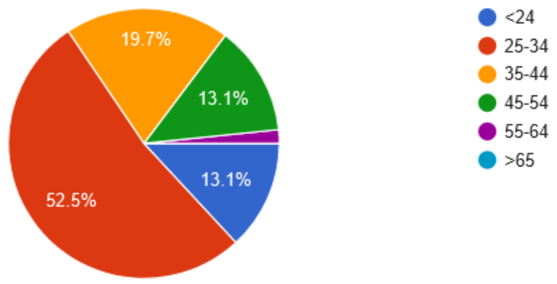

Level of education:

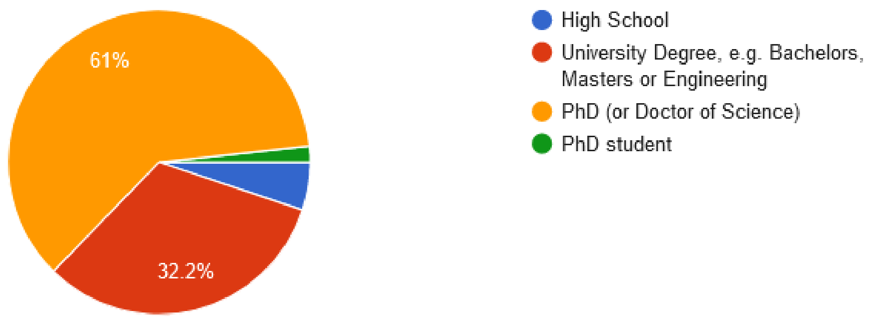

Country of residence

- Australia: 1
- Austria: 3
- Belgium: 1
- Bhutan: 1
- Brazil: 1
- Costa Rica: 1
- Egypt: 1
- Gabon: 1
- Germany: 1
- India: 16
- Mongolia: 1
- Pakistan: 1
- Romania: 3

- Russia: 2
- Taiwan: 1
- Tanzania: 1
- the Netherlands: 1
- United Kingdom: 1
- Ukraine: 25
- United States: 1

Have you participated in any past Geo-Wiki campaigns?

- No, first time: 34 (53%)
- Yes, already participated before: 30 (47%)
    - 2018: 7 (11%)
    - 2017: 23 (36%)
    - 2016: 9 (14%)
    - 2015 or earlier: 7 (11%)

Please choose the campaigns in which you participated now:

- Human Impact on Tropical Forests: 38 (59%)
- Human Impact on Temperate Forests: 37 (58%)
- Human Impact on Boreal Forests: 32 (50%)
- Follow-up campaign on disagreement locations: 19 (30%)

How did you find out information about the campaign?

- Colleagues: 34 (53%)
- Geo-Wiki Newsletter: 27 (42%)
- Social Media: 10 (16%)
- Friends and Family: 9 (14%)
- IIASA web-site: 1 (2%)

Please rank different motivations for why you (high or very high rank)

- This is a very interesting project!: 55 (86%)
- Co-authorship: 51 (80%)
- Happy to contribute to scientific research: 50 (78%)
- To join the Geo-Wiki community: 44 (69%)
- Having fun while exploring landscapes from above: 37 (58%)
- Competing against others: 27 (42%)
- Amazon voucher: 19 (30%)

Please list any additional motivations for why you participated

- Self-education, possibility to take part in scientific dialog with colleagues from other countries, importance of the nature preservation, especially forests
- To explore spatio-temporal changes in forest cover
- Cause the Importance of forest for society... biodiversity, carbon storage and water cycle....

- getting experience, curiosity, help in the project
- Gives me an overview idea of the world biomes in general and boreal forest in particular
- Gives me extra motivation for my research
- Gets extra motivation on my research
- I like to do image classification using Geo-spatial techniques.
- Self-development, interest, desire to be useful, care for nature
- Because I have high interest on investigation topics of forest biodiversity and plant communities in the case of their structure, distribution and anthropogenic dynamics.
- An interesting volunteer work and forest knowledge
- Because I have high interest on investigation topics of forest biodiversity and plant communities in the case of their structure, distribution and anthropogenic dynamics.
- Need the layer for other papers :)

Why did you stay engaged in the campaign(s)?

- I was having fun: 19 (30%)
- I was learning new things 47 (75%)
- To try to become a co-author: 50 (79%)
- To try to win an Amazon voucher: 17 (27%)
- I enjoyed the competitiveness 24 (38%)
- To learn something about crowdsourcing: 1 (2%)
- Dialog with colleagues, new scientific experience: 1 (2%)

If you stopped part-way during a campaign, what were the reasons?

- I did not have time to participate more actively: 41 (75%)
- I was not satisfied with the way my quality of work has been measured: 9 (16%)
- I realized I could no longer win co-authorship or an Amazon voucher: 6 (11%)
- It was too difficult to navigate through the pages: 2 (4%)
- The task was too difficult: 1 (2%)

Did you find the task of collecting forest specific information easy?

- Very easy: 0
- Easy: 12 (19%)
- Average: 31 (48%)
- Difficult: 20 (31%)
- Very difficult: 1(2%)

Did you use the "Ask the experts for help" button?

- Yes: 50%
- No: 50%

How helpful were the "Quick start guide" and " Examples"?

- Not needed: 0
- Rear: 4 (6%)
- In some cases: 8 (13%)
- Rather helpful: 24 (38%)
- Very helpful: 28 (44%)

How often did you analyse NDVI time series while classifying?

- Never: 20 (31%)
- Rear: 18 (28%)
- In some cases: 10 (16%)
- Rather often: 11 (17%)
- Very often: 5 (8%)

How often did you check Sentinel images?

- Never: 14 (22%)
- Rear: 17 (27%)
- In some cases: 15 (23%)
- Rather often: 12 (19%)
- Very often: 6 (9%)

How would you rate your overall experience with Human Impact on Forests?

- Poor: 1 (2%)
- Fair: 0 (0%)
- Average: 14 (22%)
- Good: 24 (38%)
- Excellent: 25 (39%)
